# Temporal multiomic modeling reveals a B-cell receptor proliferative program in chronic lymphocytic leukemia

**DOI:** 10.1038/s41375-021-01221-5

**Published:** 2021-04-08

**Authors:** Cedric Schleiss, Raphael Carapito, Luc-Matthieu Fornecker, Leslie Muller, Nicodème Paul, Ouria Tahar, Angelique Pichot, Manuela Tavian, Alina Nicolae, Laurent Miguet, Laurent Mauvieux, Raoul Herbrecht, Sarah Cianferani, Jean-Noel Freund, Christine Carapito, Myriam Maumy-Bertrand, Seiamak Bahram, Frederic Bertrand, Laurent Vallat

**Affiliations:** 1 Laboratoire d’ImmunoRhumatologie Moléculaire, INSERM UMR-S1109, LabEx Transplantex, Plateforme Genomax, Fédération de Médecine Translationnelle de Strasbourg (FMTS), Université de Strasbourg, Strasbourg, France; 2 Fédération Hospitalo-Universitaire (FHU) Omicare, Université de Strasbourg, Strasbourg, France; 3 Laboratoire d’Immunologie, Plateau Technique de Biologie, Pôle de Biologie, Nouvel Hôpital Civil, Strasbourg, France; 4 Université de Strasbourg, INSERM, IRFAC UMR-S1113, Strasbourg, France; 5 Service d’Hématologie, Institut de Cancérologie Strasbourg Europe (ICANS), Strasbourg, France; 6 Laboratoire de Spectrométrie de Masse BioOrganique, Université de Strasbourg, CNRS, IPHC, UMR 7178, Strasbourg, France; 7 Laboratoire d’Hématologie, Pôle de Biologie, Hôpital de Hautepierre, Hôpitaux Universitaires de Strasbourg, Strasbourg, France; 8 Institut de Recherche Mathématique Avancée, CNRS UMR 7501, LabEx IRMIA, Université de Strasbourg, Strasbourg, France; 9 Present Address: Fédération Hospitalo-Universitaire (FHU) Omicare, Université de Strasbourg, Strasbourg, France; 10 Present Address: Institut Charles Delaunay, ROSAS, M2S, Université de Technologie de Troyes, Troyes, France; 11 Present Address: Université de Strasbourg, INSERM, IRFAC UMR-S1113, Strasbourg, France; 12 Present Address: Laboratoire d’Hématologie, Pôle de Biologie, Hôpital de Hautepierre, Hôpitaux Universitaires de Strasbourg, Strasbourg, France

**Keywords:** Cancer genomics, Chronic lymphocytic leukaemia, Oncogenesis

## Abstract

B-cell receptor (BCR) signaling is crucial for the pathophysiology of most mature B-cell lymphomas/leukemias and has emerged as a therapeutic target whose effectiveness remains limited by the occurrence of mutations. Therefore, deciphering the cellular program activated downstream this pathway has become of paramount importance for the development of innovative therapies. Using an original ex vivo model of BCR-induced proliferation of chronic lymphocytic leukemia cells, we generated 108 temporal transcriptional and proteomic profiles from 1 h up to 4 days after BCR activation. This dataset revealed a structured temporal response composed of 13,065 transcripts and 4027 proteins, comprising a leukemic proliferative signature consisting of 430 genes and 374 proteins. Mathematical modeling of this complex cellular response further highlighted a transcriptional network driven by 14 early genes linked to proteins involved in cell proliferation. This group includes expected genes (EGR1/2, NF-kB) and genes involved in NF-kB signaling modulation (TANK, ROHF) and immune evasion (KMO, IL4I1) that have not yet been associated with leukemic cells proliferation. Our study unveils the BCR-activated proliferative genetic program in primary leukemic cells. This approach combining temporal measurements with modeling allows identifying new putative targets for innovative therapy of lymphoid malignancies and also cancers dependent on ligand–receptor interactions.

## Introduction

In B-cell lymphomas and leukemias such as marginal zone lymphoma and chronic lymphocytic leukemia (CLL), chronic antigenic activation of the B-cell antigen receptor (BCR) sustains aberrant lymphocyte behavior and uncontrolled monoclonal proliferation [1]. In physiological condition, BCR-mediated cell activation is crucial for proliferation and differentiation of lymphoid progenitors [2], as well as for recognition of pathogen-derived antigens by mature B lymphocytes. By contrast in CLL, BCR-mediated lymphocyte activation by various antigens [3], reinforced by microenvironmental factors [4], triggers aberrant cell proliferation in CLL’s proliferation centers [5]. The exploration of pathways that transfer information from BCR engagement to the nucleus revealed signaling aberrations that are reinforced by ectopic expression of the protein kinase ZAP70 in the most aggressive forms of CLL [6]. The use of specific inhibitors targeting the tumor cell survival dependency on key signaling proteins (BTK, Pi3K) [7] has proven its efficacy in clinics, however mutations in elements of these pathways ultimately lead to tumor resistance and escape. Thus, the need for alternative therapeutic approaches requires identifying novel targets. The genetic program downstream the BCR signaling cascades, namely the resulting sequential and concerted expression of multiple genes and proteins, is a promising candidate, but it still remains poorly understood.

Ex vivo models of BCR stimulation in patients’ primary CLL cells [5, 8–10] have been developed to decipher these downstream genetic programs. However, transcriptional responses analyzed at sparse time points after BCR engagement have yielded only limited insight into the complexity of the cellular response. Moreover, it is worth noting that the experimental conditions using isolated BCR activation in these previous studies led to CLL lymphocyte apoptosis instead of proliferation [11], which therefore missed the malignant proliferative output of BCR engagement observed in patients. Recently, we designed a novel ex vivo experimental setting in which BCR engagement coupled to minimal mandatory costimulating agents (CD40L, IL-4 and IL-21) recapitulate the proliferation of primary CLL cells [12].

In the present study, we used this improved ex vivo culture model to generate a unique set of 108 combined transcriptional and proteomic profiles over time after activation of human primary CLL cells. As existing analysis methods of such high dimensional datasets are limited in terms of precision to select the relevant actors of the genetic program, we have developed a mathematical approach, validated on synthetic datasets and supported by extensive simulations, allowing the selection of critical actors of the CLL’s cell response [13]. Furthermore, in order to characterize the underlying structure of the concerted temporal interactions between these actors, we refined our previously developed regression method based on linear equations [14] which has proved its capacity to handle high dimensional dataset while taking into account the inherent sparsity of biological processes [15, 16]. By using this mathematical modeling approach applied to the 108 omics points of measurement, we characterized the temporal cellular response of CLL cells to BCR activation and we identified within this response a nested and structured core proliferative program that could sustain CLL cell leukemogenesis.

## Materials and methods

### Subjects, B-cell isolation, and culture conditions

B cells from peripheral blood were obtained from six untreated CLL patients whose biological characterization, performed at the University Hospitals of Strasbourg, included flow cytometry analysis, cytogenetic with FISH, IGHV status, and TP53 mutational profile. All selected patients had a Matutes score of 5/5, unmutated IGHV, and wild-type TP53 (Table 1). This study was approved by the ethic committee (CPP Est IV) of Strasbourg University Hospitals and all patients gave written informed consent. CLL B cells were negatively selected using the RosetteSep B-cell enrichment cocktail (STEMCELL Technologies, Vancouver, Canada) and density gradient centrifugation. CLL cells (>96% CD19+/CD5+) were stained with 0.5 μM carboxyfluorescein succinimidyl ester (CFSE) (CellTrace, Thermo Fisher, Waltham, MA, USA) and were stimulated with F(ab′)2 anti-human IgM (Jackson ImmunoResearch, West Grove, PA, USA), CD40L (Enzo Life Sciences, Villeurbanne, France), IL-4 (R&D Systems-Bio-Techne, Lille, France), and IL-21 (Invitrogen, Maryland, USA) in soluble medium as described previously [12]. Cell apoptosis, evaluated using FITC-Annexin V and propidium iodide (apoptosis detection kit, BD Biosciences, San Jose, CA, USA), 4 days after BCR engagement by flow cytometry [12] (Cytomics FC500, Beckman-Coulter, Fullerton, CA, USA) showed 86–98% live cells (median: 89%) in all samples. B-cell proliferation, defined by cell division-dependent decrease in CFSE staining intensity, was monitored 6 days after BCR engagement by flow cytometry as previously described [12].

**Table 1:** Clinical and biological characteristics of CLL patients.

Sample	Sex	Age at diagnosis	IGHV status^a^	VH identity (%)	ZAP70 status^b^	CD38^c^	cytogenetic	Binet stage	Lymphocytes (G/L)	BCR response^d^
CLL-P1	F	70	UM	100	Pos	Neg	tri12	A	18	P
CLL-P2	M	67	UM	99	Pos	Neg	tri12	A	22	P
CLL-P3	F	72	UM	100	Pos	Pos	del13q	A	11	P
CLL-NP1	F	58	UM	100	Pos	Neg	0	A	28	NP
CLL-NP2	M	61	UM	100	Pos	Pos	0	A	56	NP
CLL-NP3	F	55	UM	99	Pos	Neg	del13q	A	15	NP

^a^≥ 98% of IGHV identity for defining unmutated (UM) CLL cells [52].

^b^<7 threshold of T cells/CLL cells ratio of ZAP70 mean fluorescence intensity expression for defining ZAP70-positive CLL cells [53].

^c^≥30% threshold for defining CD38-positive CLL.

^d^>25% of cell division-dependent decrease in CFSE staining intensity measured by flow cytometry at day 6 after initial B-cell receptor activation for defining proliferative (P) samples, and <20% for defining nonproliferative (NP) samples.

### Transcriptomic analysis

Before BCR engagement (T0) and at eight time points after activation (1 h, 1 h 30 min, 3 h 30 min, 6 h 30 min, 12 h, 24 h, 48 h, 96 h), 4.10^6^ cells were resuspended in 1 mL TRIzol (Sigma-Aldrich, Saint-Louis, MO, USA). Total RNA was purified using the RNeasy Mini kit (Qiagen, Hilden, Germany). After ribosomal RNA depletion, the sequencing library was prepared with the Ion Total RNA-seq kit v2 (Thermo Scientific) and the sequencing was performed on an Ion Proton sequencer with the Ion PI Hi-Q Sequencing 200 Kit (Thermo Scientific). Reads was estimated with the package HTSeq [17] and the edgeR package [18] was used to derive the reads per kilobase per million values. The transcriptomic dataset is available in GEO (GSE130385).

### Proteomic analysis

Before BCR stimulation (T0) and at eight time points after stimulation (1 h, 2 h, 4 h, 7 h, 12 h, 24 h, 48 h, 96 h), 8.10^6^ cells were resuspended in lysis buffer. Proteins were acetone precipitated and 10 µg of each sample were concentrated in a stacking gel band, in-gel reduced, alkylated, and trypsin digested. NanoLC-MS/MS analyses of extracted trypsic peptides were performed on a nanoAcquity UPLC device (Waters Corporation) coupled to a Q-Exactive Plus mass spectrometer (Thermo Scientific) operated in data dependent acquisition mode. Label-free extracted ion chromatogram-based quantification was performed using MaxQuant software (version 1.5.5.1) [19]. The proteomics dataset was deposited to the ProteomeXchange Consortium via the PRIDE partner repository (PXD013573).

### Gene expression and protein abundance analysis

Quality-based filtering of low expressed genes was performed with the HTSFilter package [20]. The selection of differentially expressed (DE) genes was made with the glmLRT and the glmTreat functions of the edgeR package [18]. Identification of temporal clusters of gene expression was performed with the HTSCluster package [21]. After quantile normalization, differential analysis of protein abundancies was made using the peptide-level robust ridge regression implemented in the MSqRob package [22].

### Clustering and network reverse engineering

After selection according to their differential expression and temporal profile, genes and proteins were divided into temporal clusters for network reverse engineering. We had to model $N$ gene or protein actors for the reverse engineering across *T* = 8 time points and for a number of *P* = 3 individuals (3 proliferative and 3 nonproliferative samples); we denote by $x_{npt}$ the observed value (gene expression or protein abundancy) of the actor $n$ for an individual $p$ at a time point $t$. For any actor of the network $n$ among the total $N$, the mathematical model was written

$$\widetilde {{x}}_{{{np}}.} = \mathop {\sum}\limits_{n^{\prime} = 1}^N {\omega _{n^{\prime} n}{\boldsymbol{F}}_{{{m}}\left( {{{n}}^{\prime} } \right){{m}}\left( {{n}} \right)}\widetilde {{x}}_{{{n}}^{\prime} {{p}}.}} + {\mathbf{\varepsilon }}_{{{np}}},\quad 1 \le p \le P.$$

In this model, $N$ is the total number of actors, $k {\,}\mapsto{\,} m(k)$ is the function that maps an actor to its time cluster, ${\boldsymbol{F}}_{{\boldsymbol{ij}}}$ is a $T$ square matrix that describes the action of the actors belonging to cluster $i$ on an actor that belongs to cluster $j$, $\omega _{kl}$ is the strength of the connection from actor k toward actor l and ${\mathbf{\varepsilon }}_{{\boldsymbol{np}}}$, and $1 \le p \le P$ is a $T$ dimensional random vector with zero mean and unit variance ${{I}}_{{T}}$.

The code written for selection of actors and reverse engineering the temporal cellular program in this study is available as an R-package (https://fbertran.github.io/Patterns/) [16].

Experimental procedure is summarized in Fig. S1 and methods are detailed in Supplementary Information.

## Results

### Identification of a structured proliferative signature in BCR stimulated CLL cells

#### Experimental design and multiomic dataset

To investigate the proliferative response of primary human CLL cells after ex vivo BCR engagement, BCR stimulation was performed on six untreated CLL samples of the most aggressive subgroup (IGHV unmutated), including three samples that proliferate and three samples that do not proliferate ex vivo within our culture conditions, the latter being used as controls (Table 1). Transcriptional (RNA-seq) and proteomic (mass spectrometry) responses of these CLL samples were determined at T0 (before stimulation) and at eight time points between 1 and 96 h after BCR-mediated cell activation, generating a total of 108 points of measurement (Fig. S1). A total of 23,348 transcripts and 4664 unique proteins were identified and quantified in the whole dataset. After quality-based filtering, 13,065 transcripts and 4027 proteins expressed at least in one of the 108 samples were retained for further analysis.

#### Unsupervised analysis identified a structured BCR response

**Fig. 1:**
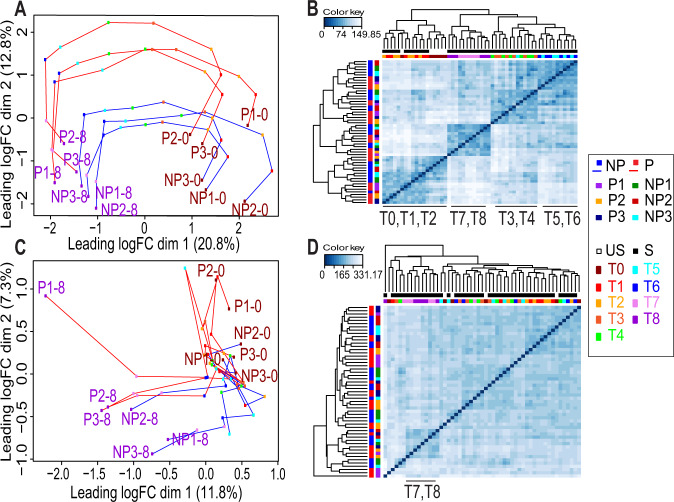
Unsupervised statistical analysis of genes and proteins expression. **A** Multidimensional scaling plot (MDS) analysis based on the expression of the 500 most expressed genes for each pairwise comparisons between the samples (among a total of 13,065 normalized gene expressions), analyzed before (T0) and at eight time points (T1–T8) after ex vivo B-cell antigen receptor activation for six chronic lymphocytic leukemia (CLL) patients (three proliferative samples (P1-3) and three control nonproliferative samples (NP1–3)). The MDS graphs were constructed from the LogFC of the expressions/abundances at different time points (T1–T8 versus T0). Each dot represents the transcriptional profile of one CLL cell sample at a specific time point. A color code represents the different time points. Successive time points of a same cell sample are linked in the graph (red line for proliferative samples and blue line for nonproliferative samples). **B** Hierarchical clustering of all samples and all time points, based on the expression of the 500 most expressed genes. Dendrograms from clustering are added to the left side and to the top of the image. The abbreviations of the times (T0–T8) represented in the different time clusters observed on the hierarchical clustering are shown at the bottom**. C** MDS analysis based on the expression of the 500 most abundant proteins for each pairwise comparisons between the samples, analyzed before and after ex vivo cell activation for the six CLL patients. Each dot represents the proteome of one CLL cell sample at a specific time point before (T0) and at eight time points (T1–T8) after cell stimulation. A color code represents the different time points. Successive time points of a same cell sample are linked in the graph. **D** Hierarchical clustering of all samples and all time points, based on the expression of the 500 most abundant proteins.

The temporal transcriptional response was explored by unsupervised multidimensional scaling which summarizes within one dot on a two-dimensional graph the 500 most expressed genes of each sample (Fig. 1A). This representation revealed the temporality of the response on the *X* axis and the proliferative status of the samples on the *Y* axis. The dots corresponding to the proliferative and nonproliferative samples were separated at T0 along the *Y* axis and they all followed a structured evolution from T1 to T8 along the *X* axis after BCR engagement. This analysis emphasized the structured nature of the transcriptional BCR response. In addition, hierarchical clustering analysis strengthened this structured nature since it identified four clusters, each made of consecutive time point of measurement (Fig. 1B). Moreover, unsupervised temporal gene expression analysis revealed clusters of genes exhibiting structured temporal patterns of expression (Fig. S2A), characterizing the transcriptional response to an exogenous stress [23, 24].

In comparison, the unsupervised proteomic analysis appeared less structured after BCR stimulation than the transcriptional one, mainly at early time points (T1–T6). However, a tendency for samples’ segregation with respect of their proliferative response was observed at later time points (T7 and T8) (Fig. 1C). This was further confirmed by hierarchical clustering (Fig. 1D). In addition, the unsupervised temporal protein expression analysis allowed identification of clusters of proteins with structured patterns of abundance modulation after stimulation (Fig. S2B).

#### The supervised analysis revealed a proliferative signature

**Fig. 2:**
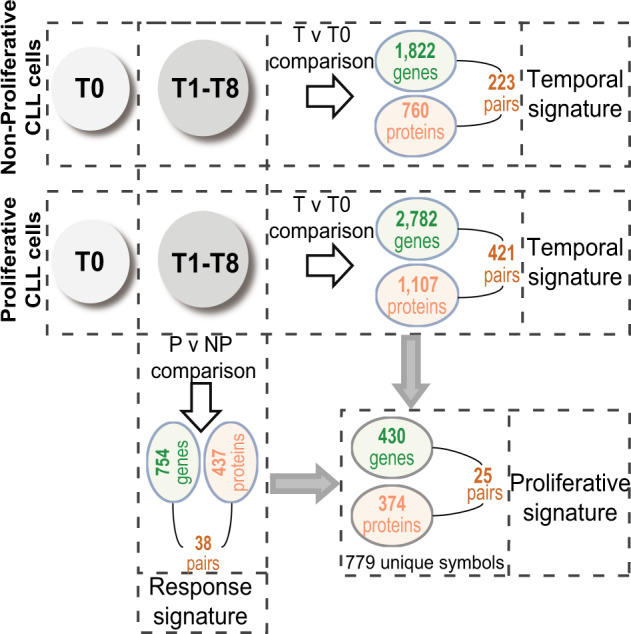
Supervised statistical analysis of genes and proteins temporal expression. Temporal signature (T versus T0 comparison, horizontally). Number of genes differentially expressed (DE) and proteins differentially abundant (DA) in proliferative (*n* = 3) and control nonproliferative CLL samples (*n* = 3) across time after cell stimulation (T1–T8), compared to initial (T0) expression/abundance (FDR < 1%). Response signature (proliferative versus nonproliferative comparison, vertically). Number of DE genes and DA proteins in proliferative CLL cells, compared to nonproliferative cells (FDR < 5%). Proliferative signature (combination of T versus T0 and proliferative versus nonproliferative comparisons). The intersection of the list of DE genes and DA proteins expressed in proliferative samples after BCR engagement compared to T0 (T versus T0), and the list of DE genes and DA proteins in the proliferative samples compared to the nonproliferative samples (proliferative versus nonproliferative) identifies the “proliferative signature” of genes and proteins specifically DE/DA after stimulation in proliferative samples.

Having evidenced the structured nature of the global CLL cell response to BCR activation, we next characterized the proliferative signature within this response, defined as the genes and proteins DE in the proliferative samples compared to the nonproliferative samples after BCR-mediated CLL cells activation. To determine this signature, we first established the list of genes and proteins significantly (FDR: <1%) up- or downregulated in the stimulated (T1–T8) versus unstimulated (T0) samples, defining a temporal signature (Fig. 2, Table S1). In the proliferative samples, 2782 DE genes and 1107 differentially abundant (DA) proteins were assigned to this temporal signature, from which 421 were pairs of common symbols (gene and corresponding protein). The nonproliferative CLL samples showed a less important temporal signature with 1822 DE genes and 760 DA proteins, from which 223 were common symbols. Secondly, we analyzed the list of genes and proteins significantly up- or downregulated (FDR: <5%) in the proliferative versus nonproliferative samples over the T1–T8 timecourse (Fig. 2, Table S1). This response signature comprised 754 DE genes and 437 DA proteins from which 38 were common symbols. The intersection of the temporal signature with the response signature of the proliferative samples showed 430 DE genes and 374 DA proteins, corresponding to 779 unique symbols, characterizing the proliferative signature after BCR engagement (Fig. 2, Table S1).

#### Strong gene-to-protein correlation within the temporal signature of the proliferative samples

**Fig. 3:**
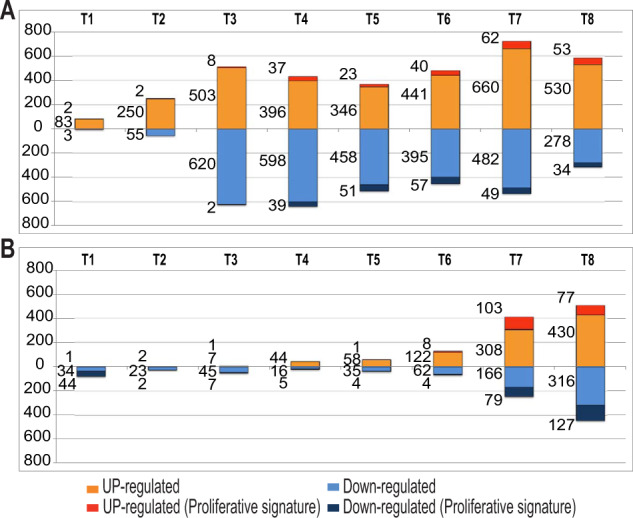
Supervised statistical analysis of genes and proteins temporal expression in proliferating CLL cells. **A** Number of DE genes and **B** number of DA proteins at each time point after cell stimulation in proliferating CLL cells. At each time point (T1–T8), the number of genes and proteins up- or downregulated (T versus T0 Log2FC) are shown in orange or blue, respectively. The number of genes and proteins specifically up- or downregulated in proliferative compared to nonproliferative cells are shown in dark orange or dark blue respectively in the graph.

**Fig. 4:**
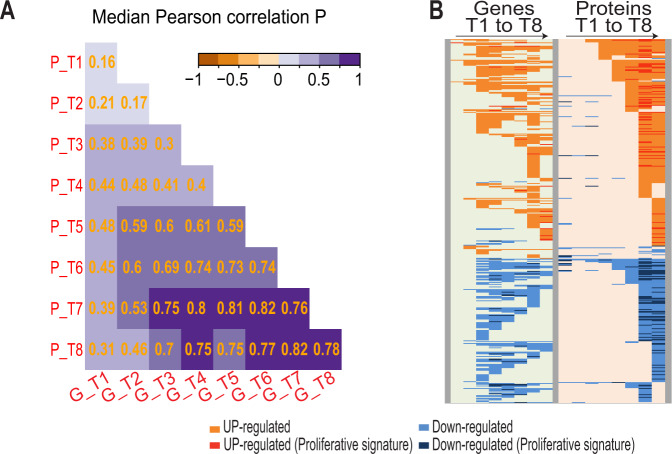
Correlation of gene expressions and protein abundancies. **A** Correlation between gene (G) and corresponding protein (P) at each time point after BCR engagement. The median value of the individual Pearson gene/protein correlation is indicated and represented with a color scale. **B** Heat map of the temporal expression/abundance of the 421 gene–protein pairs in the proliferating cells. Each line represents the temporal expression of a gene and its corresponding protein. At each time point, upregulated (T versus T0-positive Log2FC) or downregulated (T versus T0-negative Log2FC) genes and proteins are shown in red or blue, respectively.

As expected, we observed a delay between the transcriptional and the translational response after cell stimulation. Indeed, as many as 1133 genes (511 upregulated and 622 downregulated) were already DE in proliferative samples in the 3 h after BCR stimulation (Fig. 3A), whereas the proteomic modulation became obvious only after 24–48 h (Fig. 3B). Moreover, analyzing the correlation rate between gene expression and protein abundance in the set of 421 genes/proteins pairs of the temporal signature revealed a low median Pearson correlation at the initial time points (38% at 6 h) which strikingly increased up to 82% at 48–96h (Fig. 4A). This was further confirmed by the heat map of temporal expression of these 421 gene/protein pairs (Fig. 4B) showing 90% of concordance between DE genes and DA proteins expression, with a median translation delay of 6 h.

#### Functions of proteins involved in the temporal signature

We next analyzed the function of the 1107 up- or downregulated proteins of the temporal signature in the proliferative CLL cells. Biological process annotations (GO BP terms) were collected in order to calculate the number of proteins involved in each process during the timecourse after cell activation. Albeit no significant proteomic enrichment was noticed at early time point, an increase in the number of upregulated proteins related to signaling, transcriptional activity, and cell activation was observed between 7 and 24 h (T4–T6), and a second upsurge of upregulated proteins related to signaling, metabolism, transcriptional processes, cell cycle regulation, DNA replication, nuclear division, and proliferation occurred from days 2 to 4 (T7 and T8) (Fig. S3). The most represented functions during this last period were related to cell cycle regulation, DNA replication, nuclear division, and proliferation, consistent with the onset of proliferation observed in these cells after 4 days post BCR stimulation. Also, the number of proteins participating in antigen processing and peptide presentation was increased, consistent with BCR stimulation in lymphoid cells.

Looking at GO BP for downregulated proteins revealed a transiently decreased number of proteins related to signaling, metabolism, and differentiation within 1 h (T1) after cell activation, potentially reflecting a catabolism phenomenon (Fig. S3). The number of downregulated proteins remained low until 24 h (T2–T6) and no particular BP enrichment could be evidenced. However, a specific enrichment in downregulated proteins related to signaling, transcription, and cell cycle was observed at days 2–4 (T7 and T8), suggesting a negative control of these pathways at later time points.

Altogether, this multiomic approach highlights the structured nature of the temporal response to BCR stimulation in primary CLL cells, characterized by an early transcriptional component progressively relayed by a proteomic component including elements related to the onset of cell proliferation.

### Mathematical modeling of the CLL proliferative program

#### BCR response program inference in proliferative CLL cells

To model the cellular response displayed in the multiomic dataset, we used a mathematical unsupervised reverse engineering approach based on regression and system of equations that already proved its efficiency in our previous transcriptional study [15]. Temporal matrix of interactions between genes and proteins (Fig. S4A–C) was estimated by mean of penalized regression using a weighted variant of stability selection algorithm [25] in order to retain the best potential regulators of each gene or protein in the network and to determine the timing of these interactions after cell stimulation. To enhance the reverse engineering relevance [26], we imposed biological constraints by favoring links based on known transcriptional or protein–protein relationships implemented in the RegNetwork database [27]. The robustness of this inference has been ascertained by cross validation [28] and the best result was retained by linear regression estimation. Performances of the resulting model, including sensitivity, precision, predictive positive value, and *F*-score, were validated with simulated data and compared to the performance of other algorithms (Fig. S4D–F).

**Fig. 5:**
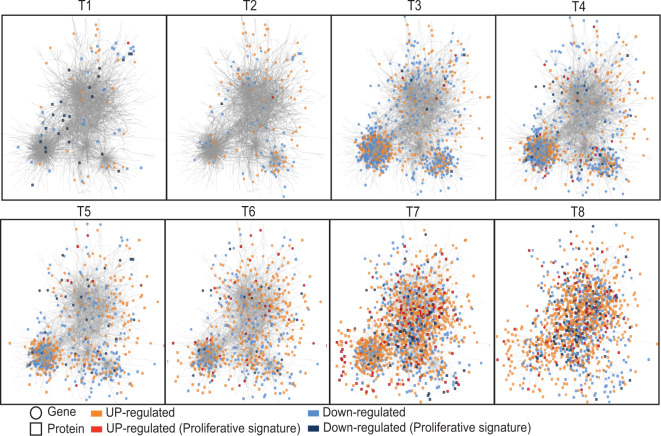
Temporal propagation in the transcriptional and proteomic network of 2167 genes and 1074 proteins induced after BCR stimulation in proliferating cells. Temporal graphical representation of statistical interactions (arrows) between genes (circle) and/or proteins (square) across time in the proliferating CLL cells after B-cell receptor stimulation. A color code represents genes and proteins differentially (DE/DA T versus T0) upregulated (orange) or downregulated (blue) at each time point after cell activation. Genes or proteins specifically up- or downregulated in proliferating cells from the proliferative signature (DE/DA T versus T0 and DE/DA P versus NP) are represented in dark orange or dark blue, respectively. Graphical representation made with Cytoscape software.

Inferring the formalized model with the temporal dataset of proliferating CLL cells identified a regulatory network of 2167 genes and 1074 proteins representing 2846 unique symbols (Table S2), among which 395 gene–protein pairs, connected by 53,131 oriented links (Fig. S5). This network exhibited a scale-free topology, where a limited number of hub genes and proteins with multiple links (12 genes and 52 proteins exhibit ≥ 10 statistical links) drove the structure of this cellular program. A detailed graphical representation allowed reconstructing the timeline of this program (Fig. 5). This temporal representation showed the DE genes and DA proteins at each time point after cell stimulation and revealed the dynamic propagation of the transcriptional and proteomic expression waves after BCR stimulation in proliferative CLL cells.

#### Deciphering a CLL proliferative program within the BCR response

Considering the ability of CLL cells to generate a proliferative response after BCR activation, we investigated whether a subnetwork sustaining cell proliferation can be identified within the above response network. To address this, we identified within the proteomic dataset of proliferating CLL cells the 267 proteins associated with the BP terms “cell cycle regulation” and “proliferation,” designed as “seeding proteins,” and their 243 connected neighbors (gene or protein) in the network (Fig. S6A). Analyzing the level of interconnection within this subgroup of proteins and genes revealed a nested subnetwork comprising 388 elements including 173 of the seeding proteins linked to 215 neighbors (Fig. S6B, Table S2). Of note, among these 388 genes and proteins elements, 31% belonged to the “proliferative signature” defined above in the supervised analysis (see also Fig. 2). This nested subnetwork could be stratified into three layers of actors (Fig. S6C).

**Fig. 6:**
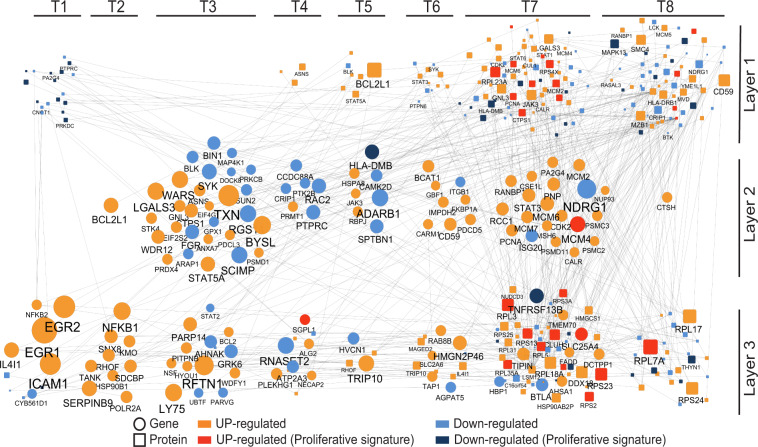
Nested temporal proliferative program induced after BCR stimulation in proliferating CLL cells. The proliferative temporal subnetwork is represented in a time ordered graph, with genes (circle) and proteins (square) represented at their first time point of differential expression after cell activation (first time DE/DA T versus T0). Genes and proteins up- or downregulated are represented in orange or blue, respectively, and size of circles and squares are proportional to fold changes (Log2FC T versus T0). The 173 seeding proteins involved in “cell cycle” or “proliferation” are grouped in the upper part of the graph (layer#1). The 71 genes coding some of these 173 proteins are represented in the middle (layer#2). The 50 genes and 94 proteins also included in this proliferative subnetwork are grouped in the lower part of the graph (layer#3).

The first layer corresponds to the 173 seeding proteins associated with cell cycle regulation or proliferation processes. These proteins segregate within two groups according to their chronological involvement during the temporal response (Fig. 6). The first temporal group comprises 18 proteins, downregulated at T1 after cell activation. These proteins are mainly involved in transcriptional repression (CNOT1, PA2G4), negative regulation of BCR signaling (INPP5D), or apoptotic process (PRKDC). The second group is made of 155 proteins whose changes occurred from T4 onward, just preceding the initiation of cell proliferation. Within this group, 85 proteins are upregulated and 70 are downregulated proteins. The upregulated proteins comprised factors involved in G1/S transition or DNA replication (e.g., PCNA, CDK2, CUL1, RANBP1, MCM), whereas downregulated ones show elements involved in signaling downstream of the BCR (BLK, BTK, LCK, SYK), potentially reflecting negative regulation mechanisms after BCR engagement.

The second layer corresponds to the 71 genes present in the subnetwork that encode proteins of the first layer (Fig. 6). Remarkably, expression modulation of these transcripts is highly correlated with the modulation of abundance of their corresponding proteins with an offset of 3–6 h.

The third layer of this subnetwork comprises 144 elements made of 50 genes and 94 proteins. At early time points (T1 and T2), only 19 elements are present of which 14 genes exhibit a strong upregulation corresponding to the very early transcriptional response to CLL cell activation. These genes encode major transcription factors (TFs) involved in the G0/G1 transition (EGR1, EGR2), in the regulation of B-cell proliferation, and differentiation after BCR activation (NFKB1, NFKB2) which are also involved in lymphomagenesis [1]. Another genes within this group encode molecules involved in signaling and NF-kB modulation (TANK, RAS homolog family member F (RHOF), syndecan-binding protein (SDCBP)), immune evasion (KMO, IL4I1, SERPINB9), and cell adhesion (ICAM1).

Remarkably, the mathematical modeling of the temporal multiomic data allowed to trace back the sequential organization of the proliferative CLL response from the protein effectors of cell proliferation at day 4 to the early molecular events induced after BCR activation.

#### Important actors of the proliferative subnetwork are missing in nonproliferative cells

We next investigated if this proliferative subnetwork composed of TFs and hub proteins is specific to the CLL proliferative program. For this purpose, we inferred with the same approach the formalized model with the temporal datasets of nonproliferative samples. This led to the identification of a regulatory network of 1399 genes and 750 proteins representing 1933 unique symbols, which were connected by 17,332 oriented links (Table S2). Functional analysis (BP terms) revealed 193 proteins associated with “cell cycle regulation” or “proliferation” process. Among them, 89% were shared with the seeding proteins displayed in the proliferative samples and corresponds to the core response program.

The search for neighbors of these 193 proteins revealed 131 elements with a nested subnetwork comprising 114 actors (37 genes and 77 proteins) (Fig. S7). Looking at this subnetwork, we identified a lower number of links and hubs in contrast to the proliferative samples (only 3 elements show ≥5 statistical links in the nonproliferative subnetwork, compared to 15 elements in the proliferative subnetwork). In addition, the nonproliferative samples exhibited a remarkable difference with the proliferative samples at the early time points (T1 and T2) where only 2 of the 14 early BCR-responsive genes were identified and those did not include the TFs EGR1, EGR2, NFKB1, and NFKB2. This observation emphasizes the critical relevance of these 14 early responsive genes in sustaining a BCR-mediated cell proliferation program in CLL cells.

## Discussion

The characterization of the cellular program sustaining CLL cells proliferation after BCR engagement is a major step to understand mature B-cell leukemogenesis with the ultimate goal of developing innovative therapies targeting the nuclear response to BCR activation instead of the cytoplasmic pathways that can be bypassed in resistant cancer cells. However, studying the proliferative program in primary CLL cells is challenging because of the difficulty to experimentally recapitulate cell proliferation ex vivo. Here, we used the recently developed culture model based on BCR engagement to induce CLL cell proliferation [12] which allowed, by a temporal multiomic approach, deciphering the dynamic and structured nature of the proliferative program triggered by BCR activation coupled to costimulating agents. For this study, special attention was paid to the similarity of the pathological characteristics among patients (untreated, IGHV unmutated, Binet A/Rai 0/1 stage), which was retrospectively attested by the absence of DE genes and DA proteins with an FDR > 5% at T0 before BCR engagement.

Our large multiomic study highlights in human primary cancer cells the coordination between the dynamic gene and protein responses after exogenous cell stimulation. So far, only few studies have addressed this relationship showing a relatively weak gene-to-protein correlation of 30–60% in yeast [29–31], murine fibroblasts, or human cancer cell lines [32, 33]. Although we showed only a 38% correlation in the first 6 h after BCR engagement, the ratio strikingly increased up to 82% at the later time points in the proliferative cells. This witnesses the progressive emergence and propagation of the organization of transcription subsequently translated into a functional protein pattern triggered by cell activation. In addition, we observed a similar median delay of 3–6 h between gene and protein expression as in yeast [29, 31]. This delay could explain the lack of temporal structure revealed by the unsupervised multidimensional scale analysis of protein abundance early after cell stimulation in contrast to the structured response displayed at the transcriptional level.

Remarkably, the proliferative subnetwork identified by modeling the response of aggressive CLL lymphocytes to BCR activation with costimulating agents comprised several genes encoding important TFs downstream the BCR signaling pathway, including genes previously identified in common in the lymphocytic response to BCR alone in healthy donors and patients with indolent or aggressive CLL [15] (Table S2). This indicates that aggressive CLL lymphocytes still retain similarities with healthy lymphocytes for their response to BCR, and is consistent with the ability of the temporal multiomic modeling approach used here to reconstruct the temporal and functional relationships from the first TFs committed 1 h after BCR engagement to proteins sustaining proliferation days after stimulation. Moreover, if we retain the 374 proteins of the proliferative signature (Fig. 2), instead of retaining the 267 proteins with a GO term of proliferation as seeding proteins, the modeling also highlights a subnetwork comprising 13 of these 14 overexpressed genes, which shows the robustness of this approach.

Among the TFs identified here in the response of aggressive CLL lymphocytes, EGR1 and EGR2 are zinc-finger TFs downstream of the Ras/Raf/MAP kinase pathway that is constitutively activated in various cancers and blood malignancies [34]. EGR1 induces survival and a proliferative response in quiescent cells and is a major driver of mature B-cell lymphomas [35]. Other upregulated genes belonging to the proliferative subnetwork include two members of NF-kB family, NFKB1 (p50) responsible for transient response after cell stimulation through the canonical NF-kB pathway and NFKB2 (p52) which is crucial for cell differentiation through the noncanonical NF-kB pathway. Via the transcriptional activation of several antiapoptotic genes, NF-kB members promote survival and proliferation of various cell types. This pathway is also crucial in B-cell leukemogenesis [1] and constitutive NF-kB activation has been described in several B-cell neoplasms [36]. Strikingly, the subnetwork also highlights genes encoding signaling proteins potentially modulating NF-kB activation, but whose implication in leukemia or lymphoma leukemogenesis has not been explored yet. For example, the TRAF family member-associated NF-KB activator (TANK) modulates NF-kB activation through binding with TRAF and TBK1 proteins [37, 38]. The RHOF, representative of the Rho GTPase family implicated in tumorigenesis by regulating cytoskeleton’s dynamic [39], has a potential role in germinal center formation [40] and has recently been involved in NF-kB regulation [41]. The SDCBP gene encodes a PDZ domain-containing protein, involved in exosome biogenesis [42] and Rho GTPase family regulation [43], and participates in NF-KB activation in melanoma [44]. The sorting nexin 8 gene, involved in endocytosis and endosomal sorting, interacts with JAK1 and IKKβ and also regulates NF-kB [45].

Furthermore, this subnetwork suggests a prominent activation of immune-evasion mechanisms of CLL cells after BCR and associated factors mediated cell proliferation activation. The genes KMO and IL4I1, respectively, involved in tryptophan catabolism [46] and germinative center formation [47] have T-cell proliferation inhibition abilities [46, 48]. This subnetwork also highlights the role of agents associated and acting in synergy with BCR activation in the ex vivo stimulation model. Among the 388 actors of this subnetwork, ten genes (BCL2L1, EGR2, FGR, ICAM1, PCNA, PRDX4, SERPINB9, STAT3, TAP1, TXN) have also been reported in the transcriptional signature of CD40L [49]. SERPINB9, a serine protease, protects cells from granzyme B associated apoptosis induced by cytotoxic T cells [50] and its expression correlates with clinical outcome of several lymphomas [51].

Noteworthy, the nonproliferative cells response did not exhibit most of the above genes, validating the composition of the core subnetwork of the BCR-mediated cell proliferation. However, comparison of the 388 actors (genes or proteins) of the proliferative subnetwork (Fig. 6) and the 114 actors of the nonproliferating subnetwork (Fig. S7) shows 60 common actors (representing 52% of the total nonproliferating actors and 15% of the proliferating subnetwork actors), constituting the core of the common response of this group of lymphocytes of the aggressive form of CLL.

In conclusion, using a large dataset of temporal transcriptional and proteomic measurements coupled with mathematical modeling, this study unveils the genetic program downstream the signaling cascade activated by the BCR engagement and triggering primary CLL cell proliferation ex vivo. Noteworthy, this program organizes around a limited number of genes and proteins whose sequential commitment drives the cellular response leading to proliferation days after cell activation. These hubs represent potential targets for the development of novel therapeutic strategies for the treatment of aggressive CLL. Beyond CLL, such an approach could be explored in other mature B and T antigen-driven malignancies and could also be extended to other cancer types dependent on ligand–receptor interactions, as for instance the hormone-dependent cancers.

## Supplementary information

Supplemental Methods

Supplemental Figures_Revised

Table S1

Table S2_Revised

## Data Availability

RNA-seq data: GEO accession GSE130385. Proteomic data: ProteomeXchange Consortium accession PXD013696. Packages availability: The Cascade package is available at https://CRAN.R-project.org/package=Cascade and https://fbertran.github.io/Cascade/. The SelectBoost package is available at https://fbertran.github.io/SelectBoost/. The Patterns package is available at https://fbertran.github.io/Patterns/.
