# Supplementary material for: Temporal multiomic modeling reveals a B-cell receptor proliferative program in chronic lymphocytic leukemia
Source: Leukemia. 2021 Apr 8;35(5):1463–74. doi: 10.1038/s41375-021-01221-5 (PMC8102193; doi:10.1038/s41375-021-01221-5)
Supplement: Supplementary file 1 — Supplemental Methods [file 41375_2021_1221_MOESM1_ESM.docx]

**Supplemental methods** (Schleiss et al.)

**Source of reagents:**

| **Product** | **Catalogue number** | **Supplier** |
| --- | --- | --- |
| CellTrace CFSE | C34554 | Invitrogen (Thermofisher) |
| Unconjugated Goat F(AB')2 Anti Human IgM FC | 109-006-129 | Jackson ImmunoResearch |
| MegaCD40L Protein | ALX-522-110-C010 | Enzo Life Sciences |
| Recombinant Human IL-4 Protein | 204-IL-010 | R&D systems |
| IL21 Recombinant Human Protein | 10447063 | Gibco (Fischer Scientific) |
| CD19-PC7 (J3-119) | IM3628 | Beckman Coulter |
| Mouse IgG1 -PC7 isotype control | 737662 | Beckman Coulter |
| CD5 FITC (BL1a) | A08932 | Beckman Coulter |
| IgG2a-FITC isotype control | A12689 | Beckman Coulter |
| Annexin V FITC | 556420 | BD Biosciences |
| propidium iodide | 556463 | BD Biosciences |

**mRNA samples preparation**

Before BCR-mediated cell activation (T0), and at eight time points after activation (1h, 1h30, 3h30, 6h30, 12h 24h, 48h, 96h), 4.10^6^ cells were collected, washed and resuspended in 1mL TRIzol (Sigma-Aldrich). Total RNA was isolated using chloroform and Phase Lock Gel tube (5 Prime) and was purified using the RNeasy Mini kit (Qiagen) according to the manufacturer's recommendation. RNA integrity was assessed with the Agilent total RNA Pico Kit on a 2100 Bioanalyzer instrument (Agilent Technologies). Ribosomal RNA was depleted with the Low Input RiboMinus™ Eukaryote System v2 kit (Thermo Fisher Scientific) following manufacturer's instructions.

**RNAseq analysis and data preprocessing**

The sequencing library was prepared with the Ion Total RNA-seq kit v2 (Thermo Fisher Scientific) according to the manufacturer's instructions. The libraries were loaded two by two at a concentration of 20 pM on an Ion PI Chip using the Ion Chef Instrument (Thermo Fisher Scientific). The sequencing was performed on an Ion Proton sequencer with the Ion PI Hi-Q Sequencing 200 Kit (Thermo Fisher Scientific). Raw sequencing data were preprocessed according to the manufacturer’s recommendations. Adapter sequences were removed and reads shorter than 18bp were excluded. Reads were then mapped against the human genome (Hg19) and a virtual reference transcriptome using TopHat2 [^1^](#_ENREF_1). Unmapped reads were mapped to the reference Hg19 using Bowtie2 in local mode [^2^](#_ENREF_2). The total reads (SAM format) was used to count reads with the default union-counting mode of the tool htseq-count of the Python package HTSeq [^3^](#_ENREF_3) (GEO accession [GSE130385](https://www.ncbi.nlm.nih.gov/geo/query/acc.cgi?acc=GSE130385)). We used the edgeR package [^4^](#_ENREF_4), to derive the reads per kilobase per million (rpkm) values from read counts and gene lengths. These rpkm values were used for differential analysis study and their Voom transformed values for model inference [^5^](#_ENREF_5). Small Nucleolar RNA (snor, 370 entries) and the microRNAs (MIR*, 1 651 entries) were filtered out.

**Proteomic samples preparation**

Before cell stimulation (T0), and at eight time points after stimulation (1h, 2h, 4h, 7h, 12h, 24h, 48h, 96h), 8.10^6^ cells were collected, washed and resuspended in 100µl of lysis buffer (Urea 8M, Thiourea 2M, Chaps 4%, Dithiothreitol (DTT) 1%, Triton 10%, TLCK 0,05%, protease inhibitors). After centrifugation (5min, 8000g), 6 volumes of glacial acetone were added to the supernatant and samples were incubated overnight at -20°C. The proteins were pelleted and 10 µg of each sample were resuspended in loading buffer (2% SDS, 0,1M DTT, 10% glycerol, 62.5 mM Tris pH 6.8) and concentrated in one stacking-gel band using a 4% SDS-PAGE gel. The gels were fixed with 45% methanol/3% acetic acid and stained with colloidal Silver Blue. Each band was excised and cut in four pieces prior to in-gel digestion. The gel pieces were washed four times with 100 μL of 75% acetonitrile (ACN) and 25% ammonium bicarbonate buffer (NH4HCO3) at 25 mM and dehydrated with 50 μL of ACN. The cysteine residues were reduced by adding 10 mM DTT for 30 min at 60°C and 30 min at room temperature, and alkylated by adding 55 mM iodoacetamide (IAA) for 20 min in the dark. The bands were then washed three times by adding 50 μL of 25 mM NH4HCO3 and 50 μL of ACN. Gel pieces were dehydrated twice with 50 μL ACN prior to enzymatic digestion. Proteins were cleaved in an adequate volume to cover the gel pieces with a modified porcine trypsin (Promega) solution at a 1:50 w/w enzyme:protein ratio. Digestion was performed overnight at 37°C. Tryptic peptides were extracted twice under agitation, first with 40 μL of 60% ACN in 0.1% formic acid (FA) for 1 h and then with 30 µL of 100% ACN for 40 min. The collected extracts were pooled, the excess of ACN was vacuum dried, and the samples were resolubilized with 25 µL of H2O/ACN/FA (98/2/0.1 v/v/v) and sonicated in ice during 10 min prior to nanoLC-MS/MS analysis.

**Mass spectrometry analysis and data preprocessing**

NanoLC-MS/MS analyses were performed on a nanoAcquity UPLC device (Waters Corporation) coupled to a Q-Exactive Plus mass spectrometer (Thermo Scientific). Peptide separation was performed on an ACQUITY UPLC BEH130 C18 column (250 mm × 75 μm with 1.7 μm diameter particles) and a Symmetry C18 precolumn (20 mm × 180 μm with 5 μm diameter particles, Waters). The solvent system consisted of 0.1% FA in water (solvent A) and 0.1% FA in ACN (solvent B). The samples were loaded into the enrichment column over 3 min at 5 μL/min with 99% of solvent A and 1% of solvent B. Peptides were eluted at 450 nL/min with the following gradient of solvent B: from 1 to 35% over 120 min and 35 to 90% over 1 min. The MS capillary voltage was set to 1.8 kV at 250°C. The system was operated in Data-Dependent Acquisition (DDA) mode with automatic switching between MS (50 ms/scan over a 300–1800 m/z range with R = 70 000) and MS/MS (100 ms/scan over a 200–2000 m/z range with R = 17 500) modes. The ten most abundant ions (intensity threshold 2 x 105) were selected on each MS spectrum for further isolation and higher energy collision dissociation (HCD) fragmentation, excluding unassigned and monocharged ions. The dynamic exclusion time was set to 60 s. Raw data collected during nanoLC-MS/MS were processed using MaxQuant (version 1.5.5.1) [^6^](#_ENREF_6). Peaks were assigned with the Andromeda search engine with trypsin/P specificity. The database used for the searches contained all human entries extracted from UniProtKB-SwissProt including canonical sequences and isoforms (2016, sept. ; 42 145 entries). The minimum peptide length required was seven amino acids and a maximum of one trypsin missed cleavage was allowed. The precursor mass tolerance was set to 20 ppm for the first search and 4.5 ppm for the main search. The fragment ion mass tolerance was set to 20 ppm. Methionine oxidation was set as a variable modification and carbamidomethylation of cysteines as a fixed modification. The maximum false discovery rate was 1% at peptide and protein levels with the use of a decoy strategy. The “match between runs” option was used. The dataset was made of 53 measurements of 50 503 peptides (6 subjects x 9 time points, outlying sample CLL-NP3_24h was excluded) and we used the data preprocessing pipeline recommended by the MSqRob package [^7^](#_ENREF_7). The data were log2 transformed and we applied quantile normalization. We filtered peptides that could be assessed as contaminant and we removed proteins that were only identified by peptides carrying one or more modification sites. Gene symbols were retrieved for all protein groups, whenever possible, from the UniProt database (The UniProt Consortium 2017 [^8^](#_ENREF_8), using the proteins API [^9^](#_ENREF_9). The complete proteomics dataset was deposited to the ProteomeXchange Consortium via the PRIDE partner repository with the dataset identifier PXD013573.

**Gene expression Analysis**

Selection of differentially expressed (DE) genes. The TREAT test conducts genewise statistical tests for a given coefficient or contrast relative to a specified fold-change threshold. First, separate group analysis was performed to search for genes significantly DE, with glmLRT or glmTreat, at the 5% or 1% FDR level at a given time (Ti, i>0) versus control (T0=0) in each of the proliferative (P) and non-proliferative (NP) groups (temporal signature). Second, we searched for DE genes between each groups (response signature). glmLRT and glmTreat (whenever possible) detect genes that respond differently in the NP and P groups at a given time or over all time points (with respect to control time values).

Cluster selections of genes. We performed a single run of HTSCluster looking up to K = 1, …, 35 clusters, using the Trimmed Means of M-values (TMM) normalization [^10^](#_ENREF_10), and the splitting small-EM strategy, an approach similar to that described by Papastamoulis [^11^](#_ENREF_11). In HTSCluster, model selection may be performed using the DDSE calibration for slope heuristics, Djump calibration for slope heuristics, Bayesian Information Criterion, and Integrated Completed Likelihood criterion. When the slope heuristics approach may be applied, we used the capushe package [^12^](#_ENREF_12), to provide diagnostic plots for this slope heuristics in order to ensure that sufficiently complex models have been considered.

**Proteomic Data Analysis**

Choosing the FDR procedure. According to calibration plots, p-values distributions were not standard and hence the use of the cp4p package [^13^](#_ENREF_13) to perform more involved FDR correction was required. Almost all the choices of the FDR control method provided by the cp4p package led to the same numbers of DA proteins. We selected the two-stage Benjamini and Hochberg procedure [^14^](#_ENREF_14) because it can limit the FDR to a given level and was set to 0.05 for the DA analysis. The differential analysis was made using the test.contrast_adjust function of the MSqRob package [^7^](#_ENREF_7). MSqRob implements a peptide-level robust ridge regression that improves estimation, sensitivity, and speciﬁcity in data-dependent quantitative label-free shotgun proteomics.

**Gene and protein selection for network reverse engineering**

To merge the gene and protein datasets, we matched the gene and protein groups names. Protein groups that featured multiple gene names were manually curated. The selection of the actors of the networks was made in three steps:

- Base selection = Treat 1% FDR DE genes for any of the 24 tests and 1% FDR DA protein groups for any of the 24 tests. Those 24 tests are the 8 Ti vs T0 tests for each of the two groups P, NP and the between group NP v P analysis.
- Enrichment 1 = NP v P 5% DE genes or DA protein groups for any of the 24 tests (without FDR correction for the 24 tests)
- Enrichment 2 = Based on temporal profiles expected after an exogeneous cell activation.

More precisely, a gene could be selected by the profile based enrichment if Its DE ranking is less than 200 and it features a single peak at a single time, or a single peak for two consecutive times, or a single peak for three consecutive times.

The DE ranking of gene, is its ranking among the other genes with respect to its p-value at a given test. A gene with a DE ranking equal to 1 features the smallest p-value for the test whereas a gene with the highest DE ranking possible, features the largest p-value for the test. We chose 200, a rather high value, as the max DE ranking value in order to perform a large profile based enrichment of the selection. The number of unique gene ID in the final selection is 5 733. Obviously, genes expressions or proteins abundancies must not be constant (within a given group either NP or P) to be included in the reverse engineering algorithm since we need to assess their predictive value on other gene expressions or protein abundancies. As a consequence, we had to filter out 125 protein abundancies. Any of the proteins abundancies that were removed were constant in both of the groups.

**Clustering of genes and proteins**

We first adjusted the range of the gene expressions and of the protein abundancies in order to improve the similarities between the genes and proteins groups values. To rescale abundancies’ distributions to the ranges of the expressions’ distributions, we used a multiplying factor: the 50% Trimmed Mean of the ratios of ranges of the abundancies divided by the range of the expressions for every Gene Name for which those two values were available.

To cluster genes and protein  we applied Fuzzy c−means clustering (FCM) [15](#_ENREF_15), [16](#_ENREF_16) using the Euclidean distance.

1. Methodology of the clustering.

We used two runs of the FCM methodology, detailed in section 2 below, to get the 62 final clusters of the genes (41 clusters) and proteins (21 clusters) datasets.

First, using FCM, we clustered the subset of genes and proteins, for which both gene expressions and proteins abundances were measured, according to those genes expressions and those protein abundancies, which resulted in 21 clusters. We directly used those 21 clusters to cluster the proteins, since gene expressions were missing for only a few proteins.

Yet, there were hundreds of genes expressions without protein abundance data. To tackle this issue, we performed an additional FCM run and clustered all the genes expressions into 20 clusters using only the gene expression data.

For the genes that may be clustered in the first run (genes and proteins data) and in the second run (genes without proteins data), we retained the clustered found using both gene and protein data since it is likely to be more accurate. Hence, the genes were split into 21+20 clusters and the proteins into the 21 very same clusters as the 21 genes clusters. In addition, this type of clustering was likely to help to simplify the upcoming network inference since it required to model how genes and proteins clusters interact.

2. Methodology for an FCM.

Even if Mfuzz package, that implements FCM, was first developed to cluster microarray data, the algorithms of the Mfuzz package, as claimed by its authors on their webpage (<http://mfuzz.sysbiolab.eu>), can also be applied to other types of data such as proteins abundances or RNAseq datasets.

We chose to perform the FCM cluster analysis using the Euclidean distance. As result, the expression values of genes were standardized to have a mean value of zero and a standard deviation of one. This ensures that vectors of genes with similar changes in expression are close in Euclidean space, as recommended by Futschik and colleagues [15](#_ENREF_15). To perform FCM, two parameters have to be specified: the number of clusters c and the FCM parameter m.

The FCM parameter m was estimated using the m estimate function of the Mfuzz package that implements the algorithm from Schwammle et al. [17](#_ENREF_17). To determine the number of clusters, we used the minimum centroid distance as a cluster validity index since for an optimal cluster number, we may see a ‘drop’ of minimum centroid distance when plotted versus a range of cluster number and a slower decrease of the minimum centroid distance for higher cluster number. Again, more information and some examples can be found in the study of Schwammle and Jensen (2010).

**Network reverse engineering**

Then we designed groups for both the NP and P network inferences in the following way:

1. For the 3 707 gene expression data of the gene ID with gene expression data only, we used the previously derived 20 clusters, numbered from 1 to 20. The minimum size of a group is 68.

2. For the 2 015 gene expression data of the gene ID with both gene expression and protein abundancies data, we used the previously derived 21 clusters, numbered from 21 to 41. The minimum size of a group is 44.

3. For the 2 025 protein abundancies data of the gene ID with protein abundancies data (and for 2,015 of them gene expression data), we used the previously derived 21 clusters, numbered from 42 to 62. We had to infer, using random forest imputation and the mice R package [^18^](#_ENREF_18) the group membership values for the 10 proteins that only featured protein abundancies. The minimum size of a group is 44.

We will denote by G2P the action of a cluster of genes expressions on a cluster of protein abundancies, G2G the action of a cluster of genes expressions on a cluster of genes expressions, P2G the action of a cluster of protein abundancies on a cluster of genes expressions and P2P the action of a cluster of protein abundancies on a cluster of protein abundancies (Supplementary Fig. 4A).

For any given cluster $i$ among those 62 clusters, we set, for all of them and by analyzing the time course profiles of the cluster members, the first timepoint, denoted by $O_{i}$, for which it might begin to have an effect on the other actors of the networks.

In order to take into account relevant biological knowledge, we designed a weighted inference. The algorithm aims to reveal the links between the actors of the network (genes or proteins). A weight can favor or disfavor such a link. It ranges from 0, always in, to $+\infty$, always exclude; a unit weight being neutral.

Information on regulators, targets, as well as confidence (High, Low, Medium) and evidence (Experimental, Predicted), are provided by RegNetwork (341 207 links) [^19^](#_ENREF_19). The weight values were modulated according to the actual confidence and evidence values. We chose several thresholds for the weights and matched them with the uncertainty of the biological knowledge of a given action of an actor of the network on another:

always=0<+++<++<+<1<-<+Inf=exclude

The weights also take several biological or modelling assumptions into account:

- If both gene expressions and protein abundancies were measured for a given gene ID, only the protein abundancy measurement must be used to infer its effect in the network.
- No actions within a cluster. The members of a given cluster (of genes or proteins) cannot be used to infer the expressions of the abundancies of other members of the same cluster. It makes since members of the same cluster share similar time profiles.
- No loop action (either for a gene on itself or for a protein on itself)

Such a weight is view as a penalty factor in the penalized regression model: it is a number that multiplies the lambda value in the minimization problem to allow differential shrinkage ([^20^](#_ENREF_20), equation 1 p.3). If equal to 0, it implies no shrinkage, and that variable is always included in the model. Default is 1 for all variables. Infinity means that the variable is excluded from the model. Note that the weights are rescaled to sum to the number of variables.

The core of the statistical model combined a $F$ matrix (a square matrix of size 8*62=496) that models the time dependent effects between clusters and a ω matrix (a square matrix of size 7 747) that captures a non-time dependent link (the possibility of an action) between the actors of the network. More precisely, a cell $F_{ij}$ (itself a square matrix of size T) of the $F$ matrix models the effect of cluster $i$ on cluster $j$. Notice that if an actor of the network $n_{0}$belongs to cluster $i$ and an actor of the network n belongs to cluster $j$, the matrix $F_{ij}$allows the link between the actors $n_{0}$and $n$ to be time dependent. To enforce temporal causality, we used the two following time constraints:

- A cluster $i$ cannot affect another cluster $j$, if $C_{i}\geq C_{j}$: this ensures that a cluster $i$ cannot affect a cluster $j$ if the first action time $C_{j}$ of cluster $j$ precedes the first action time of cluster $i$. Hence, if $C_{i}\geq C_{j}$, then the $F_{ij}$cell of the $F$ matrices is set to 0 ($F_{ij}$=0). Supplementary Fig. 4 B-C shows nonzero $F_{ij}$matrices for the P and NP network reverse engineering. These are the same for these data but could have happened to be different.
- If $C_{i}<C_{j}$, then the $F_{ij}$ matrix is a lower triangular square matrix of size T. Its shape is chosen so that the measurement of an actor of the network at time $t_{k}$ can influence another the measurement of an actor of the network at time $t_{k_{0}}$ if and only if $k<k_{0}$ (Supplementary Fig. 4C). The only exception being the G2P case of the action of a cluster of genes $i$ for which we also measured the proteins ($21\leq i\leq41)$ on its protein cluster (the cluster of the proteins abundancies with the same Gene ID numbered $i+21$). In that case we choose an almost diagonal matrix (Supplementary Fig. 4B). Been able to easily switch between those matrices is one of the main reasons that accounts for creating the same clusters for both genes and proteins when possible.

Hence, contrary to what was previously assumed in Vallat et al. 2013 and Jung et al. 2014 [^21^](#_ENREF_21)^,^ [^22^](#_ENREF_22), the size of the $F_{ij}$ was increased in order to cope with the G2P actions that are modelled at the same timepoint and the sub-diagonals and the diagonal of matrices F were no longer supposed to be constant since the experimental timepoints were no longer equally distributed throughout the experiment (the times differences range from 30 minutes to 2 days). A constant value on a subdiagonal of the $F_{ij}$ matrix would mean that the intensity of the effect of an actor on another one only depends on the time lag between the measurements. Nevertheless, we assumed that for similar time steps, the interactions should depend only on time index differences (i.e. time lag) rather than absolute time index. Hence some parts of the diagonal and sub-diagonal of the $F_{ij}$ matrices are constant.

We have selected $N=7 747$ actors for the reverse engineering across $T=8$ timepoints and for $P=3$ individuals; we denote by $x_{npt}$ observed value (gene expression of protein abundancy) of the actor $n$ for an individual $p$ at timepoint $t$. For any actor of the network $n$ among the $N=7 747$ ones, the mathematical model was written:

$${\tilde{\boldsymbol{x}}}_{\boldsymbol{np}\mathbf{.}}=\sum_{n^{'}=1}^{N} \omega_{n'n}\boldsymbol{F}_{\boldsymbol{m}\left( \boldsymbol{n}^{\mathbf{'}} \right)\boldsymbol{m}\left( \boldsymbol{n} \right)}{\tilde{\boldsymbol{x}}}_{\boldsymbol{n}^{\mathbf{'}}\boldsymbol{p}\mathbf{.}}+\boldsymbol{\varepsilon}_{\boldsymbol{np}}, 1\leq p\leq P.$$

In this model, $N$ is the total number of actors, $k \mapsto m(k)$ is the function that maps an actor to its time-cluster, $\boldsymbol{F}_{\boldsymbol{ij}}$ is a $T$ square matrix that describes the action of the actors belonging to cluster $i$ on an actor that belongs to cluster $j$, $\omega_{kl}$ is the strength of the connection from actor k towards actor l and $\boldsymbol{\varepsilon}_{\boldsymbol{np}}$, $1\leq p\leq P$ is a $T$ dimensional random vector with zero mean and unit variance $\boldsymbol{I}_{\boldsymbol{T}}$. So, in this model ${\tilde{\boldsymbol{x}}}_{\boldsymbol{n}\mathbf{..}}$ is the regulated actor and $\tilde{x}_{n_{0}..}$, $1\leq n_{0}\neq n\leq7 747$, are the regulators ($n_{0}\neq n$ to not allow self-regulation). It is known that genes or proteins tend to be regulated by few genes or proteins whereas a given gene or protein can regulate many other genes or proteins. Hence we chose to carry out the fitting of the model using penalized regression. In addition, in order to select only the more relevant and stable regulators for given actor, we used a weighted variant of stability selection [^23^](#_ENREF_23) combined with nonnegative least squares and a coordinate ascent approach by alternatively supposing the $\boldsymbol{F}_{\boldsymbol{ij}}$ matrices known or the $\boldsymbol{\omega}$ matrix known. To get a more robust result, the estimation of the ${\hat{\boldsymbol{F}}}_{\boldsymbol{ij}}$ matrices was done several times by ‘leave one subject out’ cross-validation. Furthermore, to avoid computational issues, after each step of the algorithm the new $\boldsymbol{F}_{\boldsymbol{ij}}$ matrices were chosen as a linear combination between the $\boldsymbol{F}_{\boldsymbol{ij}}$ matrices estimated at the end of the preceding step and the $\boldsymbol{F}_{\boldsymbol{ij}}$ matrices that were estimated at this step using non negative least squares. The result of the estimation process was threefold: (1) a connectivity network described by the nonzero elements of $\hat{\boldsymbol{\omega}}$, if $\hat{\omega}_{n'n}\neq0$ means that an action of $n'$ on $n$ was detected, (2) any ${\hat{\boldsymbol{F}}}_{\boldsymbol{ij}}$ matrix catches if there is an effect of cluster $i$ on cluster $j$ and at which time(s) those effects arise, and (3) the evolution through time of the action of actor $n'$ on actor $n$ can be derived by computing the product ${\hat{\omega}_{n'n}\hat{\boldsymbol{F}}}_{\boldsymbol{m}\left( \boldsymbol{n}^{\mathbf{'}} \right)\boldsymbol{m}\left( \boldsymbol{n} \right)}$ and by combining the $\boldsymbol{\omega}$ and $\boldsymbol{F}_{\boldsymbol{ij}}$ (code available in the Patterns R package [^24^](#_ENREF_24)).

**Data simulation and algorithm validation**

To simulate the measurements of the actors based on a regulatory network, we designed an algorithm that is inspired by the preferential attachment [^25^](#_ENREF_25). Then, we adapted it to temporal nested networks. We then used our cascade network based model to make some simulations, using Laplace laws to set the values of the measurements of the actors at the first time point.

The results of simulated data were then compared with the performances of our previous Cascade algorithm (Cascade package [^21^](#_ENREF_21)^,^ [^22^](#_ENREF_22)), its non-weighted, properly weighted and incorrectly weighted Lasso-versions (Patterns package [^24^](#_ENREF_24)) and with the performances of a non-weighted and weighted Stability Selection-version algorithm [^26^](#_ENREF_26) (Supplementary Fig. 4 D-F). We also compared these results with the performances of a weighted and non-weighted version of the SelectBoost algorithm (SelectBoost Package, results available online on the package website [^27^](#_ENREF_27)). These results showed that correctly weighted models achieved a higher sensibility, PPV and F-score than the other ones (especially Cascade) and demonstrated the performances of this modelling approach.

**References supplemental methods**

1. Kim D, Pertea G, Trapnell C, Pimentel H, Kelley R, Salzberg SL. TopHat2: accurate alignment of transcriptomes in the presence of insertions, deletions and gene fusions. *Genome Biol* 2013 Apr 25; **14**(4)**:** R36.

2. Langmead B, Salzberg SL. Fast gapped-read alignment with Bowtie 2. *Nat Methods* 2012 Mar 4; **9**(4)**:** 357-359.

3. Anders S, Pyl PT, Huber W. HTSeq--a Python framework to work with high-throughput sequencing data. *Bioinformatics* 2015 Jan 15; **31**(2)**:** 166-169.

4. McCarthy DJ, Chen Y, Smyth GK. Differential expression analysis of multifactor RNA-Seq experiments with respect to biological variation. *Nucleic Acids Res* 2012 May; **40**(10)**:** 4288-4297.

5. Law CW, Chen Y, Shi W, Smyth GK. voom: Precision weights unlock linear model analysis tools for RNA-seq read counts. *Genome Biol* 2014 Feb 3; **15**(2)**:** R29.

6. Cox J, Hein MY, Luber CA, Paron I, Nagaraj N, Mann M. Accurate proteome-wide label-free quantification by delayed normalization and maximal peptide ratio extraction, termed MaxLFQ. *Mol Cell Proteomics* 2014 Sep; **13**(9)**:** 2513-2526.

7. Goeminne LJ, Gevaert K, Clement L. Peptide-level Robust Ridge Regression Improves Estimation, Sensitivity, and Specificity in Data-dependent Quantitative Label-free Shotgun Proteomics. *Mol Cell Proteomics* 2016 Feb; **15**(2)**:** 657-668.

8. Chen C, Huang H, Wu CH. Protein Bioinformatics Databases and Resources. *Methods Mol Biol* 2017; **1558:** 3-39.

9. Nightingale A, Antunes R, Alpi E, Bursteinas B, Gonzales L, Liu W*, et al.* The Proteins API: accessing key integrated protein and genome information. *Nucleic Acids Res* 2017 Jul 3; **45**(W1)**:** W539-W544.

10. Robinson MD, Oshlack A. A scaling normalization method for differential expression analysis of RNA-seq data. *Genome Biol* 2010; **11**(3)**:** R25.

11. Papastamoulis P, Martin-Magniette ML, Maugis-Rabusseau C. On the estimation of mixtures of Poisson regression models with large number of components. *Computational Statistics & Data Analysis* 2016; **93:** 97-106.

12. Arlot S, Brault V, Baudry JP, Maugis C, Michel B. capushe: CAlibrating Penalities Using Slope HEuristics. version 1.1.1. *R package* 2016.

13. Giai Gianetto Q, Combes F, Ramus C, Bruley C, Coute Y, Burger T. Calibration plot for proteomics: A graphical tool to visually check the assumptions underlying FDR control in quantitative experiments. *Proteomics* 2016 Jan; **16**(1)**:** 29-32.

14. Benjamini Y, Krieger AM, Yekutieli D. Adaptive linear step-up procedures that control the false dicovery rate. *Biometrika* 2006; **93**(3)**:** 491-507.

15. Futschik ME, Carlisle B. Noise-robust soft clustering of gene expression time-course data. *J Bioinform Comput Biol* 2005 Aug; **3**(4)**:** 965-988.

16. Kumar L, M EF. Mfuzz: a software package for soft clustering of microarray data. *Bioinformation* 2007 May 20; **2**(1)**:** 5-7.

17. Schwammle V, Jensen ON. A simple and fast method to determine the parameters for fuzzy c-means cluster analysis. *Bioinformatics* 2010 Nov 15; **26**(22)**:** 2841-2848.

18. Van Buuren S, Groothuis-Oudshoorn K. mice: Multivariate Imputation by Chained Equations in R. *Journal of Statistical Software* 2011; **45**(3)**:** 1-67.

19. Liu ZP, Wu C, Miao H, Wu H. RegNetwork: an integrated database of transcriptional and post-transcriptional regulatory networks in human and mouse. *Database (Oxford)* 2015; **2015**.

20. Friedman J, Hastie T, Tibshirani R. Regularization paths for generalized linear models via coordinate descent. *Journal of statistical software* 2010; **33**(1)**:** 1.

21. Vallat L, Kemper CA, Jung N, Maumy-Bertrand M, Bertrand F, Meyer N*, et al.* Reverse-engineering the genetic circuitry of a cancer cell with predicted intervention in chronic lymphocytic leukemia. *Proceedings of the National Academy of Sciences of the United States of America* 2013 Jan 8; **110**(2)**:** 459-464.

22. Jung N, Bertrand F, Bahram S, Vallat L, Maumy-Bertrand M. Cascade: a R package to study, predict and simulate the diffusion of a signal through a temporal gene network. *Bioinformatics* 2014 Feb 15; **30**(4)**:** 571-573.

23. Meinshausen N, Bühlmann P. Stability selection. *Journal of the Royal Statistical Society: series B (Statistical methodology)* 2010; **72**(4)**:** 417-473.

24. Bertrand F, Maumy-Bertrand M. Patterns: patterned networks reverse engineering. *R package* 2019.

25. Barabasi AL, Oltvai ZN. Network biology: understanding the cell's functional organization. *Nat Rev Genet* 2004 Feb; **5**(2)**:** 101-113.

26. Meinshausen N, Bühlmann P. Stability selection. *Journal of the Royal Statistical Society: Series B (Statistical Methodology)* 2010; **72:** 417-473.

27. Bertrand F, Maumy-Bertrand M, Jung N, Aouadi I. SelectBoost : a General Algorithm to Enhance the Performance of Variable Selection Methods in Correlated Datasets. version1.3.0. *R package* 2019.
