# Supplementary material for: Temporal multiomic modeling reveals a B-cell receptor proliferative program in chronic lymphocytic leukemia
Source: Leukemia. 2021 Apr 8;35(5):1463–74. doi: 10.1038/s41375-021-01221-5 (PMC8102193; doi:10.1038/s41375-021-01221-5)
Supplement: Supplementary file 2 — Supplemental Figures_Revised [file 41375_2021_1221_MOESM2_ESM.pdf]

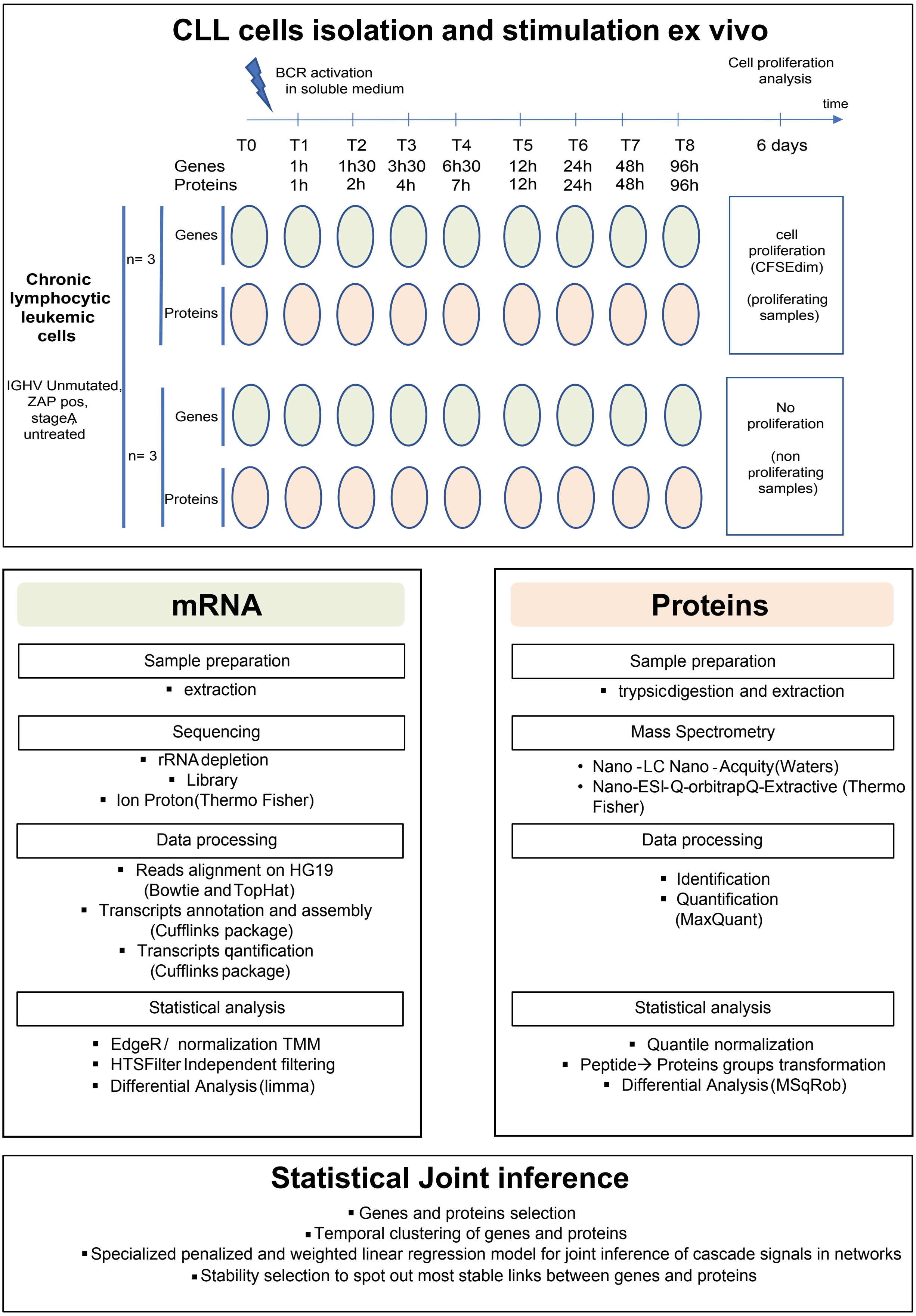

Supplementary Figure 1. Experimental design and methods outline.
Top panel. Three proliferative and three non-proliferative CLL samples have been included. BCR engagement in soluble medium was performed at T0 and cell samples were collected immediately before BCR engagement at T0 and at 8 time points after cell stimulation for gene expression (RNAseq) and protein abundance (mass spectrometry) measurements. At day six, cell proliferation has been quantified by flow cytometry after initial staining with CFSE. Bottom panel. Summary of the processing used for mRNA expression quantification, protein abundance quantification and mathematical analysis.

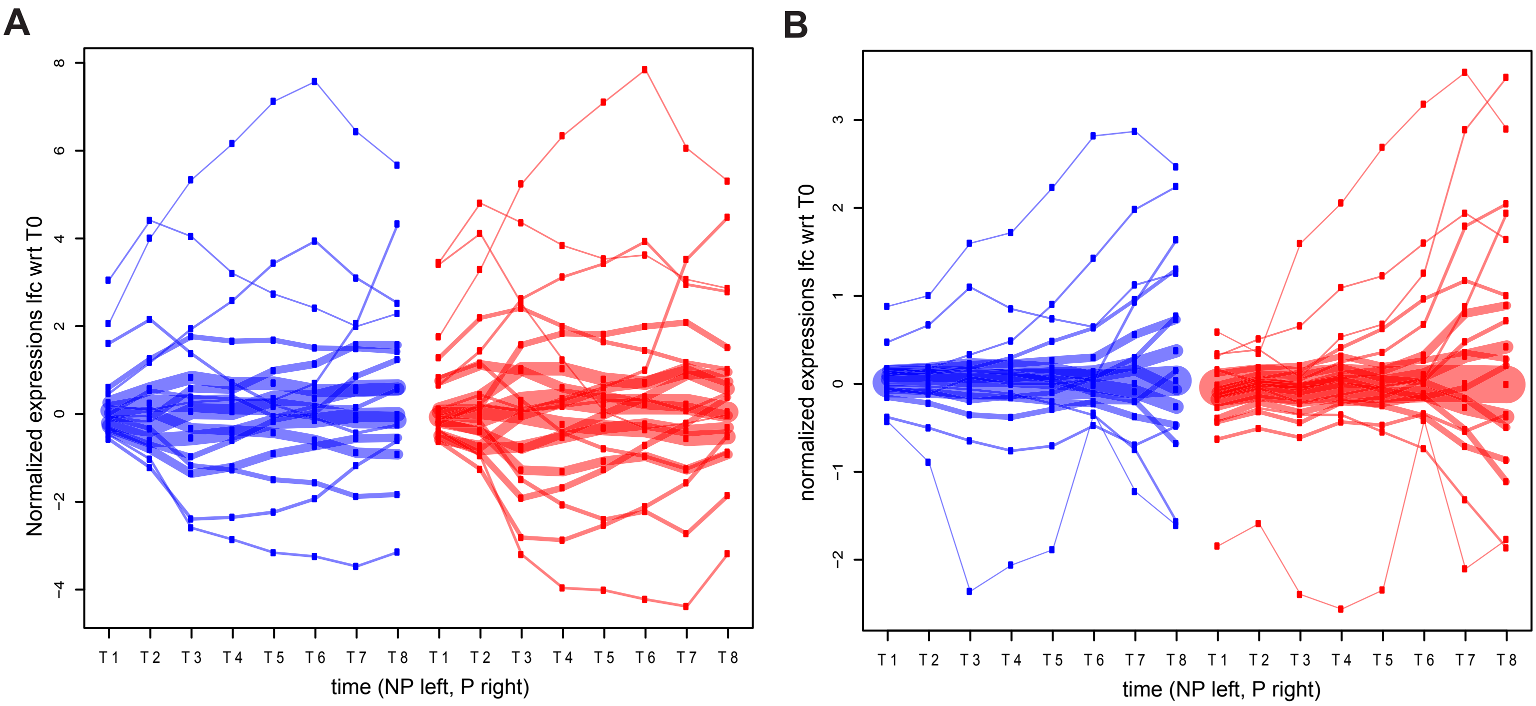

Supplementary Figure 2.

A Unsupervised temporal cluster of gene expression. Gene expression clustering of all proliferative (P, red) and non-proliferative (NP, blue) samples was unsupervised and performed separately. Median P or NP gene expression were used for a distinct representation of non-proliferative temporal cluster of gene expression or proliferative temporal cluster. The width of a line correlates with the size of the corresponding cluster.

B Unsupervised temporal cluster of protein abundance. Same as (A) for the protein abundance clustering of the proliferative (P, red) and non-proliferative (NP, blue) samples.

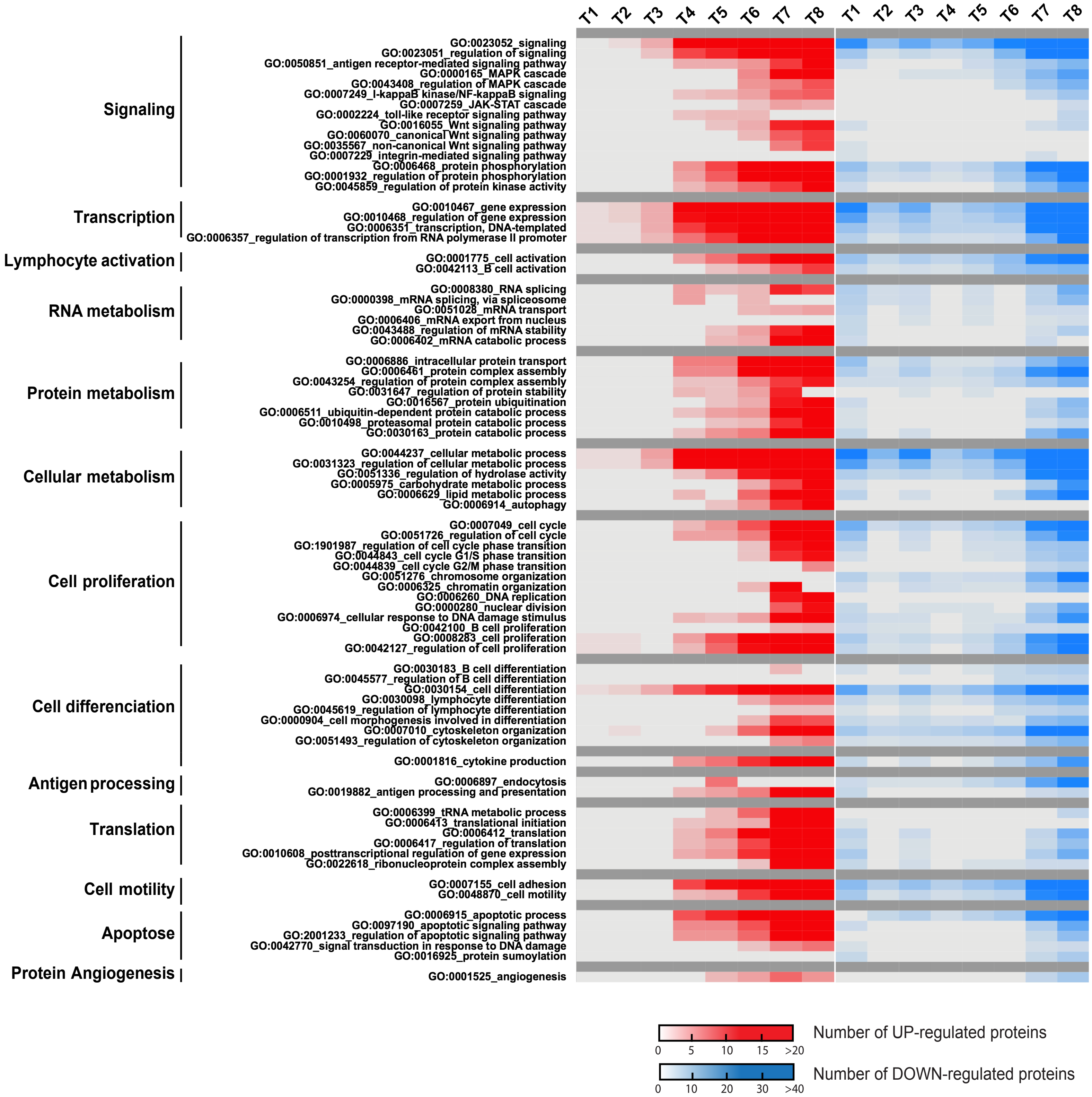

Supplementary Figure 3. Temporal representation of biological functions (GO BP terms) of up- and down-regulated proteins in proliferating CLL samples.

The heat map shows with a color code, the number of proteins differentially up-regulated (red) or down-regulated (blue) assigned to a particular Gene Ontology (GO) function, at each time point (T1 to T8) after cell stimulation of the proliferating samples. Functional enrichment of biological processes (GO BP terms) has been analyses using DAVID on the 1,107 differentially up- or down-regulated proteins in the proliferating cell samples.

A

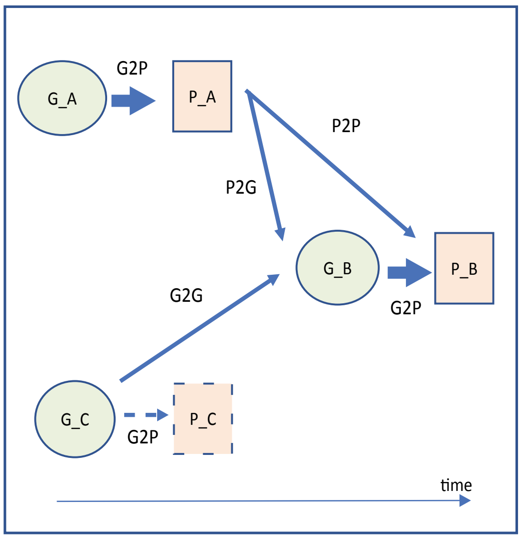

D E F

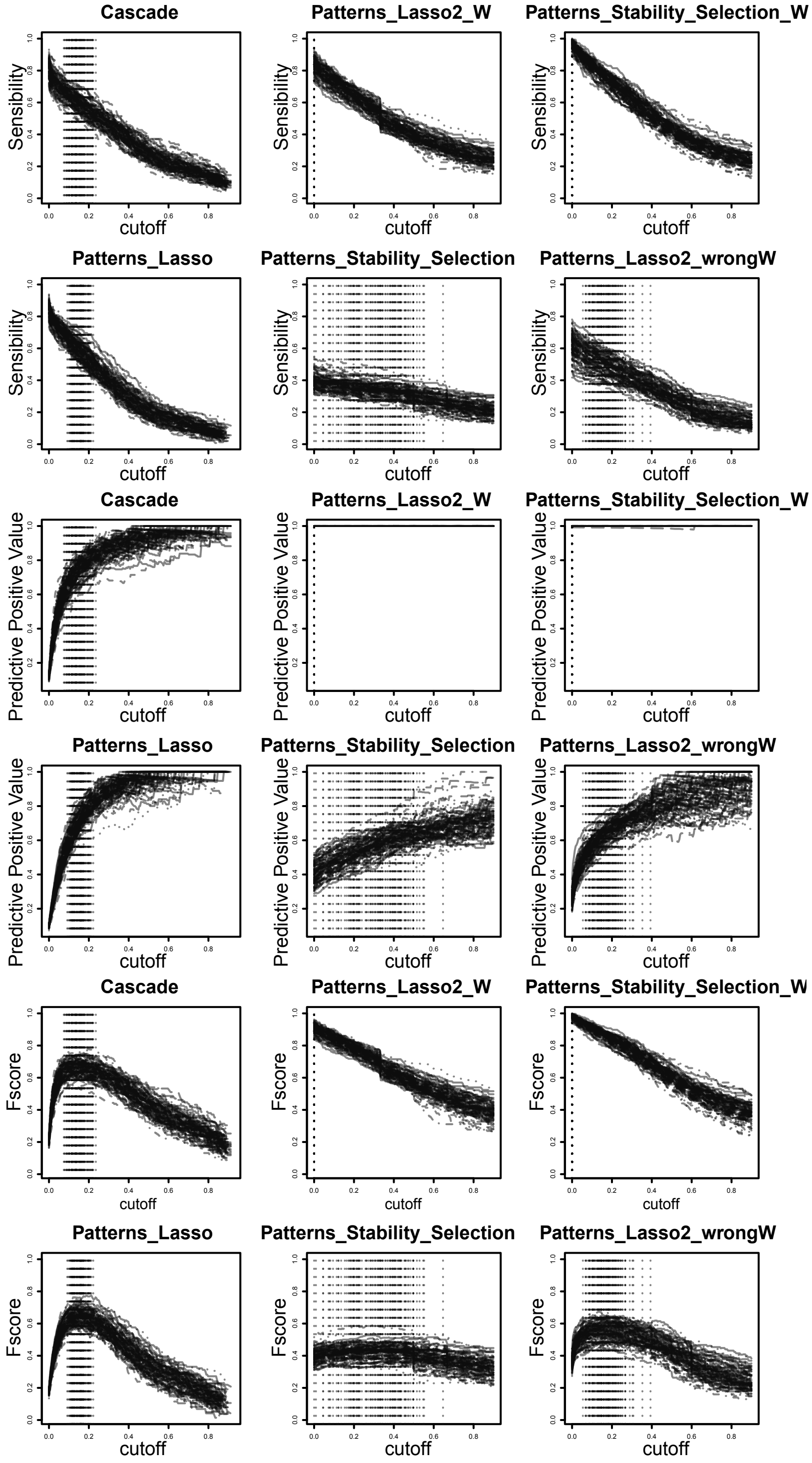

B F matrix for a G2P action

	1	2	3	4	5	6	7	8
1	p_{ij}^F	0	0	0	0	0	0	0
2	q_{ij}^F	r_{ij}^F	0	0	0	0	0	0
3	0	0	r_{ij}^F	0	0	0	0	0
4	0	0	0	r_{ij}^F	0	0	0	0
5	0	0	0	0	r_{ij}^F	0	0	0
6	0	0	0	0	0	r_{ij}^F	0	0
7	0	0	0	0	0	0	r_{ij}^F	0
8	0	0	0	0	0	0	0	r_{ij}^F

C F matrix for a G2G, P2G, P2P action

	1	2	3	4	5	6	7	8
1	0	0	0	0	0	0	0	0
2	a_{ij}^F	0	0	0	0	0	0	0
3	b_{ij}^F	a_{ij}^F	0	0	0	0	0	0
4	c_{ij}^F	b_{ij}^F	a_{ij}^F	0	0	0	0	0
5	d_{ij}^F	c_{ij}^F	b_{ij}^F	a_{ij}^F	0	0	0	0
6	0	e_{ij}^F	f_{ij}^F	i_{ij}^F	k_{ij}^F	0	0	0
7	0	0	0	j_{ij}^F	l_{ij}^F	m_{ij}^F	0	0
8	0	0	0	0	0	n_{ij}^F	o_{ij}^F	0

Supplementary Figure 4. Model formalization and validation.

A Genes and proteins are represented by circles and squares, respectively. Genes or proteins differentially expressed at least at one of the time point after cell stimulation are represented with a full lined circle or square. Genes or proteins not measured are represented with a dotted line. Authorized links between genes and/or proteins in the formalized model are represented by arrows (gene to protein (G2P), gene to gene (G2G), protein to gene (P2G), protein to protein (P2P)). Weighted links between genes and proteins with the same gene symbol (after translation of protein accession numbers in gene symbols (e.g. gene_A and protein_A) are represented in bold. Links between a protein with transcriptional factor activity and its known gene target (e.g. gene_B, or the corresponding protein_B if the gene_B is not measured in our experiment) are also weighted according to database information (e.g. RegNetwork). Matrix of temporal interactions shows authorized links between couple of time points for G2P (**B**) and for G2G, P2G and P2P (**C**). Each letter symbolizes a weight for a particular temporal link. To test our model, we analyzed the sensitivity (**D**), predictive positive value (**E**) and F-score (**F**) with Cascade algorithm, a non-weighted , an appropriately weighted and an incorrectly weighted version of our algorithm, a non-weighted stability selection based version of our algorithm (see methods). To simulate the measurements of the actors based on a regulatory network, we designed an algorithm that is inspired by the preferential attachment. Then, we adapted it to temporal nested networks. We then used our cascade network based model to make some simulations, using Laplace laws to set the values of the measurements of the actors at the first time point (see methods).

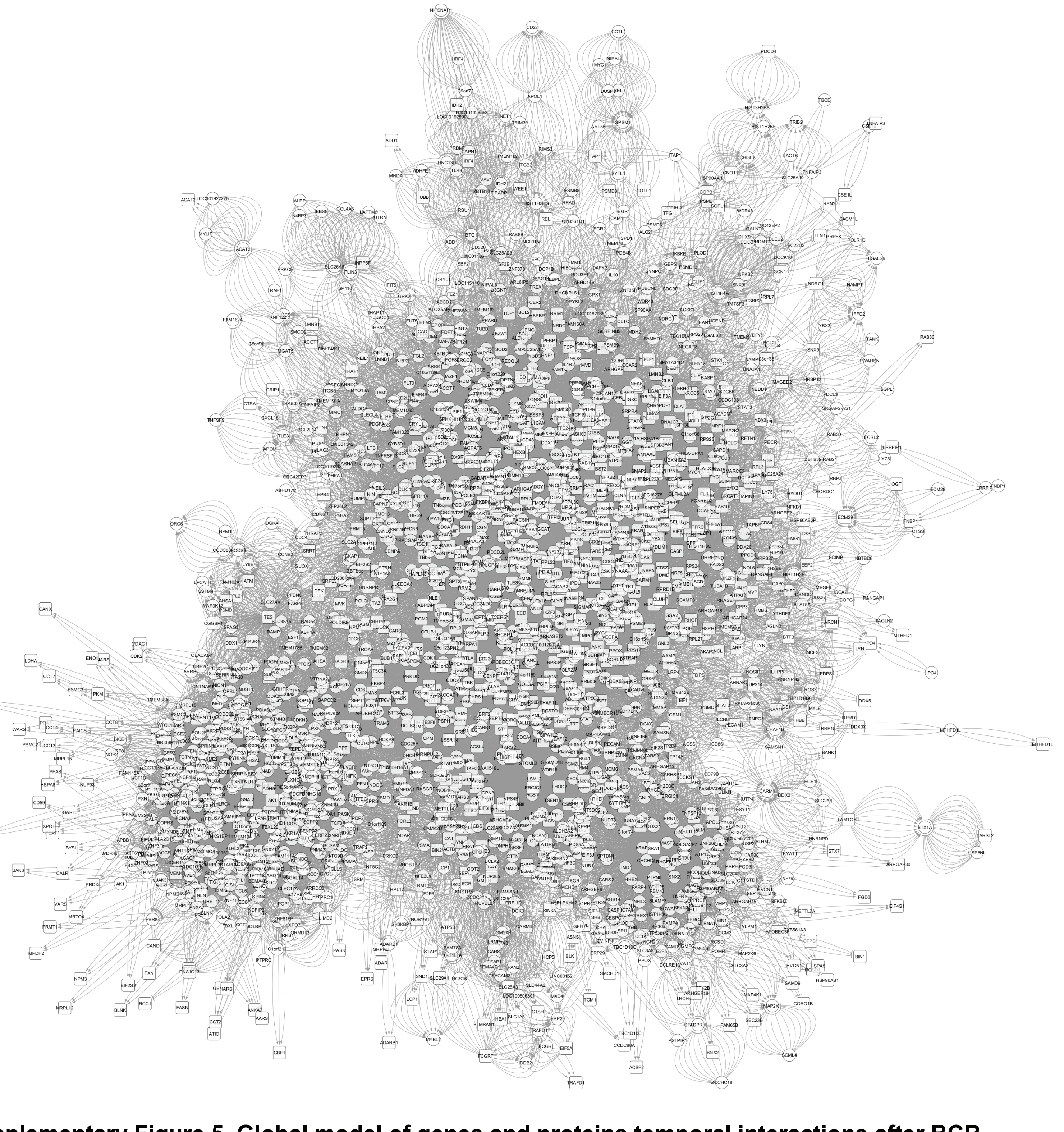

Supplementary Figure 5. Global model of genes and proteins temporal interactions after BCR stimulation in proliferating CLL samples.

Visualization of the joint inference of statistical interactions between genes and proteins differentially expressed at least at one of the time point (T v T0) after cell stimulation in proliferating cells. Circles represent genes, squares represent proteins and edges represent inferred links between genes and /or proteins across time. The graphical representation (generated with Cytoscape software) includes 2,167 genes and 1,074 proteins (for a total of 2,846 unique symbols). Potential interactions (represented in the graph when omega ≥ .01, see methods section) between genes and/or proteins expression over time are represented by time-directed arrows.

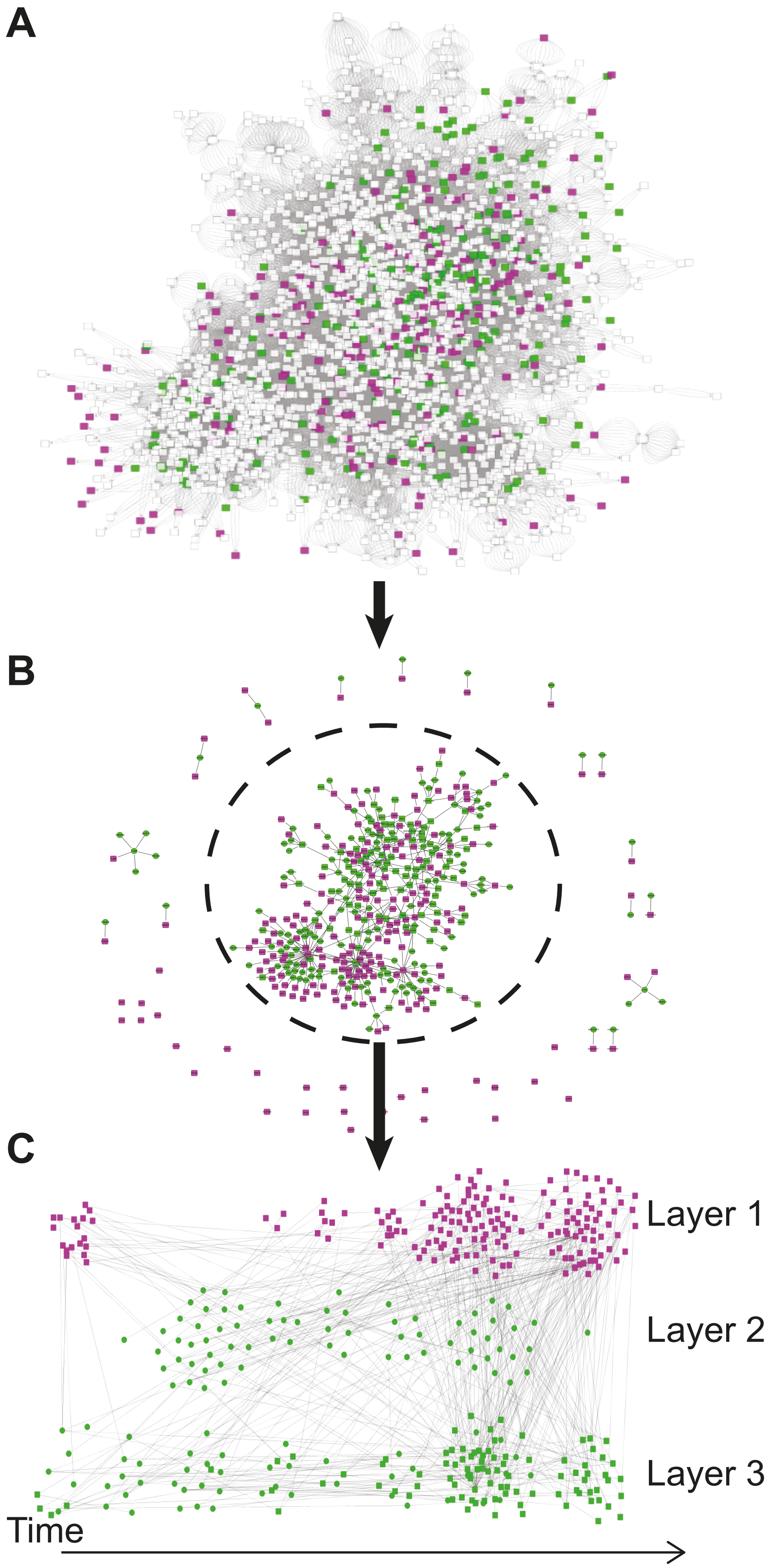

Supplementary Figure 6. A CLL proliferative program nested within the BCR-response program in proliferative CLL-cells.

A Search for protein associated with BP terms "cell-cycle regulation" or "proliferation" identified 267 seeding proteins (purple) in the global model of genes-proteins temporal interactions after BCR stimulation in proliferative CLL-cells. These seeding proteins are connected to 243 neighbors in the model (green). **B** These 267 seeding proteins and 243 neighbors organized into a major nested sub-network made of 173 of the seeding proteins and 215 neighbors. C Temporal representation of the nested sub-network, organized within three layers of actors.

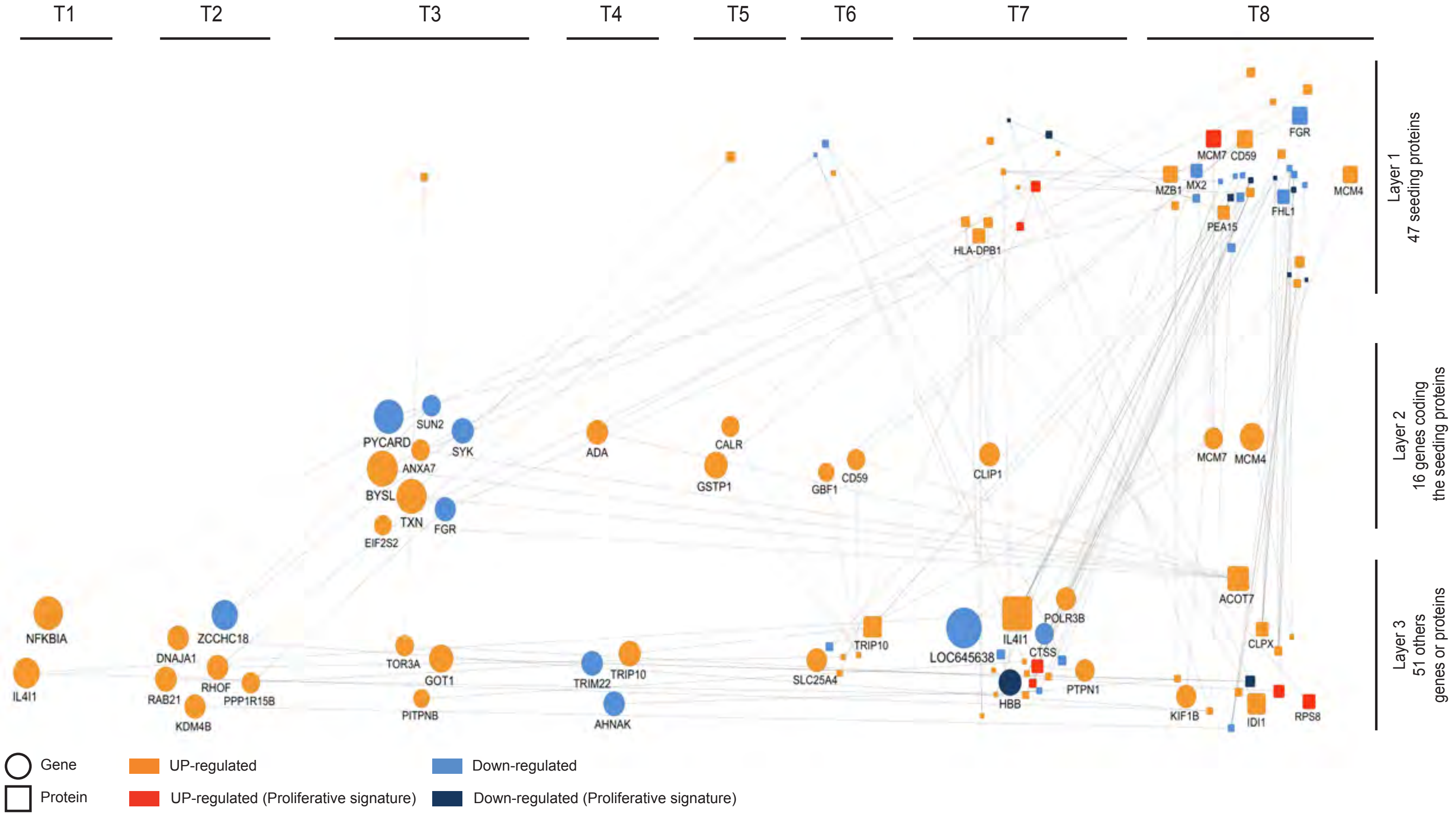

Supplementary Figure 7. Non proliferating sub-network

Search for protein associated with BP terms “cell-cycle regulation” or “proliferation” identified 193 seeding proteins in the global model of genes-proteins temporal interactions after BCR stimulation in non-proliferative CLL-cells. These seeding proteins and neighbors organized into a major nested sub-network made of 47 proteins and 67 neighbors. Temporal representation of the nested sub-network, organized within three layers of actors (generated with Cytoscape software).
